# A comparative analysis of blastoid models through single-cell transcriptomics

**DOI:** 10.1016/j.isci.2024.111122

**Published:** 2024-10-11

**Authors:** Ali Balubaid, Samhan Alsolami, Narsis A. Kiani, David Gomez-Cabrero, Mo Li, Jesper Tegner

**Affiliations:** 1 Biological and Environmental Science and Engineering Division, King Abdullah University of Science and Technology (KAUST), Thuwal 23955-6900, Saudi Arabia; 2 Algorithmic Dynamic Lab, Department of Oncology and Pathology, Karolinska Institute, Stockholm, Sweden; 3 Unit of Computational Medicine, Department of Medicine, Center for Molecular Medicine, Karolinska Institutet, Karolinska University Hospital, L8:05, SE-171 76 Stockholm, Sweden; 4 Translational Bioinformatics Unit, Navarrabiomed, Complejo Hospitalario de Navarra (CHN), Universidad Pu'blica de Navarra (UPNA), IdiSNA, Pamplona, Spain; 5 Computer, Electrical and Mathematical Sciences and Engineering Division, King Abdullah University of Science and Technology (KAUST), Thuwal 23955-6900, Saudi Arabia; 6 Science for Life Laboratory, Tomtebodavagen 23A, SE-17165 Solna, Sweden

**Keywords:** Stem cells research, Developmental biology, Transcriptomics

## Abstract

Pluripotent-stem-cell-derived blastocyst-like structures (blastoids) offer insights into early human embryogenesis (5–10 days post-fertilization). The similarity between blastoids and human blastocysts remains uncertain. To investigate, we evaluated single-cell RNA sequencing (scRNAseq) data from seven blastoid models, comparing them to peri-implantation blastocysts. We quantified cell-type composition, transcriptomic overlap, lineage profiles, and developmental propensities for primary (epiblast, primitive endoderm, trophectoderm) and potential lineages (amnion, extravillous cytotrophoblasts, syncytial trophoblasts). Blastoids from extended pluripotent stem cells (EPSCs) are distinct from those from naive pluripotent stem cells (nPSCs), which cluster closer to natural blastocysts. EPSC-blastoids show a higher composition of primitive endoderm cells and ambiguous cells with notable endoderm signatures. Starting cell lines' scRNAseq analysis reveals higher heterogeneity in nPSCs and prevalent amnionic signatures in EPSCs. These findings suggest gene expression heterogeneity in founding cells influences blastoid lineage differentiation, aiding protocol optimization for better human embryogenesis models.

## Highlights

• Blastoids are categorized as balanced, EPI_ICM enriched, or PE enriched
• EPSC blastoids are PE enriched, and nPSC blastoids are EPI_ICM enriched
• EPSCs show a higher propensity for off-target cell fates
• Cellular heterogeneity is both a driver and a challenge in blastoid formation

## Graphical abstract

**Figure:**
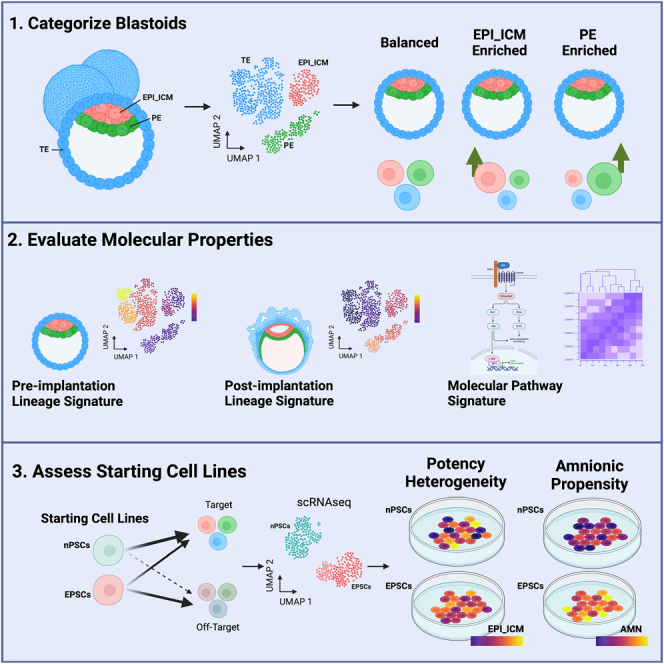

## Introduction

Understanding the mechanisms of human embryology is a significant aim for scientific and medical reasons. However, the ethical limitations have constrained the progress in this field. Recent advancements in stem cell culture and assembloid technologies have allowed the *in vitro* development of multiple blastocyst-like models, generally termed “blastoids.”^1^^,^^2^^,^^3^^,^^4^^,^^5^^,^^6^ These structures resemble natural blastocysts in morphology, presenting a sphere covered by a single-cell epithelial layer of trophectoderm-like cells enveloping a clumped population resembling the inner cell mass and a cavity corresponding to the blastocoel.^1^^,^^2^^,^^3^^,^^4^^,^^5^^,^^6^ Like how induced pluripotent stem cells (iPSCs), somatic cells reprogrammed to a pluripotent state, bypassed the ethical bottleneck, blastoids offer an opportunity to explore human embryogenesis pre-/peri-/post-implantation and cell ontogeny *in vitro*, eliciting significant impacts on researching embryonic developmental anomalies and human fertility.^7^

Different blastoid models are built through various methods (Figure S1), including those using human naive pluripotent stem cells (nPSCs),^1^^,^^2^^,^^6^ cells captured in the pre-implantation pluripotent state, iPSCs,^2^ reprogrammed fibroblasts,^3^ extended pluripotent stem cells (EPSCs), cells with extra-embryonic and embryonic developmental potentials, with single-step and multi-step induction protocols,^4^^,^^5^ as well as different culture conditions (Figure S1). Also, blastoids were generated using complex media that modulate signaling pathways with growth factors and small-molecule inhibitors^6^ (Figure S1). The success rates of these blastoid models differ, with success defined by the capture of hallmark features of the blastocyst stage. While morphological success is essential, ensuring molecular recapitulation of endogenous profiles is equally crucial. Molecular fidelity is primarily assessed through immunofluorescence techniques targeting cell-type-specific markers and single-cell RNA sequencing (scRNAseq) for transcriptomic profiling.

scRNAseq data, the quantification of RNA expressed from genes in individual cells by sequencing, provides a high-resolution view of cellular composition, enabling the exploration of lineages and cellular dynamics.^8^ Moreover, comparing different blastoid models allows for identifying molecular differences influenced by the choice of cell lines and culturing methods, even when morphological characteristics appear identical. One such example is a recent report that suggested that blastoid models’ trophectoderm (TE) cells observed in blastoids are more amnion (AMN)-like than TE.^9^ Additionally, the juxtaposition between different single-cell datasets has the potential to identify the subtle variations that are not noticeable through independent observation. However, a lack of cross-study comparative analysis remains, limiting our understanding of the factors underlying blastoid quality. We anticipate that concurrent examination of natural blastocysts may expose inherent limitations of current blastoid models and unveil opportunities to enhance their fidelity. Furthermore, investigating these differences could offer valuable insights into the most promising blastoid models for future research topics.

One source of variation among blastoid models is cell-type composition. Multiple cell types have been identified in human blastocyst studies,^1^^,^^10^^,^^11^ including the TE, making the outer layer, and the inner cell mass (ICM), a clump of cells within the blastocyst cavity. The ICM later gives rise to defined epiblast (EPI) and primitive endoderm (PE) populations, which give rise to the embryo proper and the yolk sac, respectively. After implantation, the TEs give rise to syncytial trophoblasts (STBs) and cytotrophoblasts (CTBs), which eventually give rise to the extravillous cytotrophoblasts (EVTs), forming the extraembryonic tissues anchoring the embryo to the uterine wall.^12^^,^^13^ In contrast, EPI gives rise to the AMN in the initial stages and the many other embryonic tissues that come after. By identifying these cells and their marker signals, an in-depth investigation of the biomolecular profiles of blastoid models is possible.

This report focuses on evaluating the biomolecular profiles contrasting different blastoid models to natural blastocysts using transcriptomic profiling at single-cell resolution. scRNAseq has become ubiquitous in biomedical studies and provides a detailed description of the cellular heterogeneity and composition within the sequenced samples. However, some caveats are associated with scRNAseq data due to the multiple technical steps involved in acquiring the data, such as variation in library preparation, amplification bias, differences in sequencing platforms, and batch effects.^14^ We take a systematic approach to preprocessing raw data to minimize bioinformatic-related technical biases.^14^ After mapping and quality control filtering, we annotate and evaluate cell-type composition. We then ask: (1) What are the differences in cell-type composition between the blastoid models and blastocysts? (2) Are the cell-type identities identical between the different datasets? (3) How different or similar are the transcriptomic identities between blastoids and blastocysts? To what degree do their cells overlap in the same transcriptomic landscape, and to what degree are they comparable? (4) What are the drivers of the heterogeneity of the different blastoid models? How do they explain the observed blastoid properties? By probing these questions, we seek a deeper understanding of how the blastoid models are different from natural blastocysts. By disentangling and highlighting various relevant dimensions influencing blastoid variability, we identify founding cell lines as one of the fundamental drivers of blastoid fidelity, effectively presenting the significant drivers of blastoid model heterogeneity and the potential paths toward enhanced blastocyst modeling.

## Results

Our results explore the similarity of cell-type composition and cell-type identity for the different blastoid models and blastocysts. We also investigate the transcriptional similarity in a shared transcriptomic landscape. Next, we develop an analysis through cluster distribution and defined lineage module scores, revealing a distinctive transcriptomic pattern for EPSC-based models relative to nPSC-based models. Therefore, we perform scRNAseq of the two main types of founding cells—EPSCs and nPSCs—and explore the underlying biases concerning the markers and signatures of interest.

### Primary cell lineages composition informs blastoid subtypes

**Figure 1:**
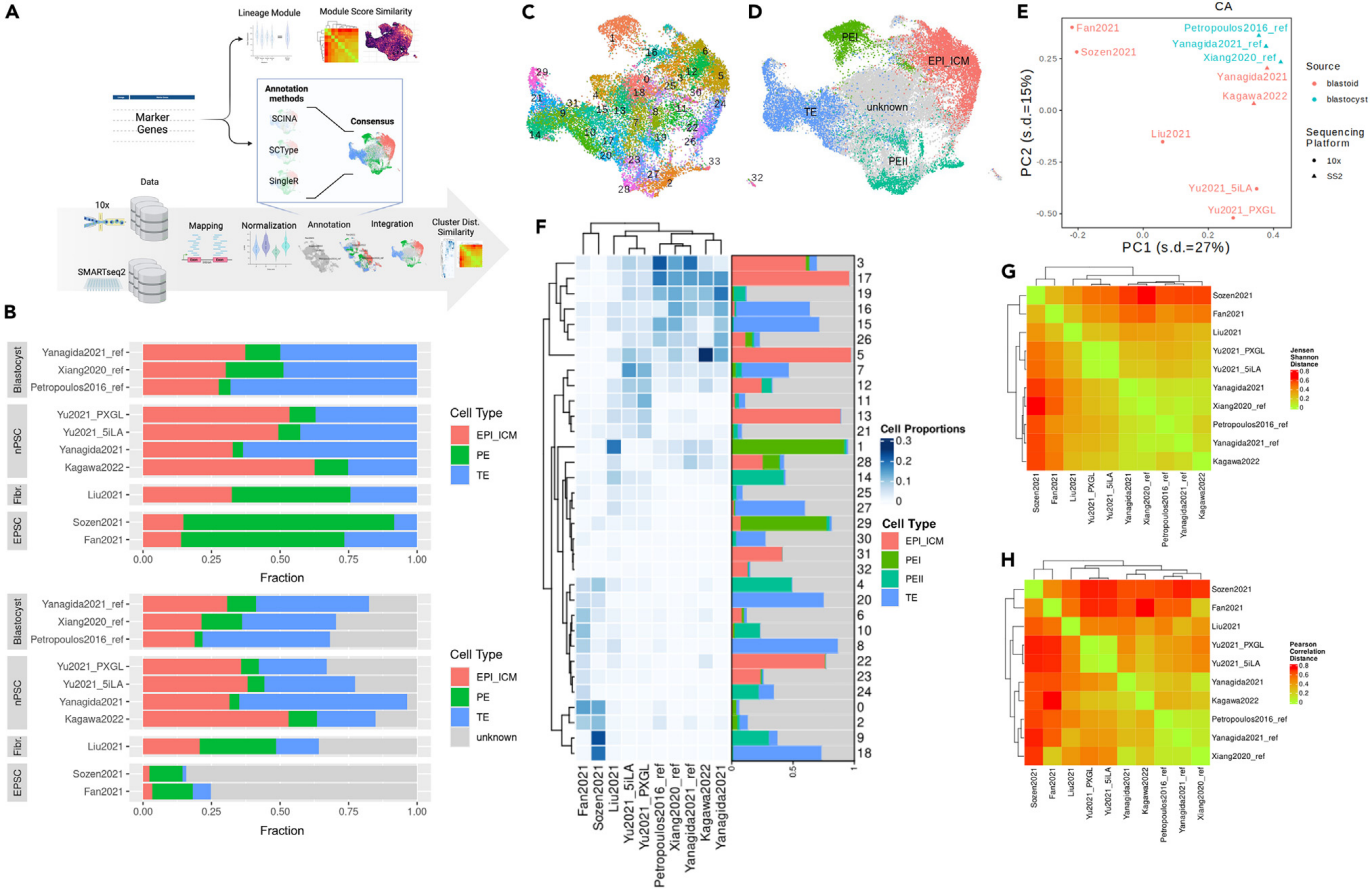
Cell type and transcriptomic profile composition analysis (A) Schematic shows an overview of our analysis based on annotation, lineage module expression, and integration cluster distribution similarity. (B) Overview of cell-type composition across datasets, illustrated with a hierarchical clustering dendrogram. (C) UMAP visualization of the integrated datasets identified by cluster. (D) UMAP visualization of the integrated datasets identified by cell type. (E) PCA of the distribution of dataset clusters, categorized by source (reference or non-reference) and sequencing technology (10x Genomics or SMART-Seq2). (F) Representation of dataset-specific cell abundance across integrated clusters, annotated with the cell-type composition within each cluster, post-integration, arranged according to hierarchical clustering. (G) Evaluation of cluster distribution similarity using the Jensen-Shannon Distance (JSD). (H) Analysis identical to (E), employing Pearson correlation distance (PCD) for similarity measurement.

We first inspect the different blastocysts and blastoid models to evaluate their cell-type composition. This pertains to asking about the differences in cell-type composition between the blastoid models and blastocysts. We first annotate the cells using the consensus between three automatic annotation methods, as shown in Figures 1A and S3A–S3D (see more in STAR Methods). We cluster the datasets by cell-type proportions using the newly assigned annotations in Figure 1B. A significant association is observed between the primary cell-type (i.e., EPI_ICM, PE, TE) composition and the source cell types (i.e., blastocyst, nPSC, EPSC, fibroblast) for both cases including only the annotated cells (χ2 statistic: 4494.5, df = 6, *p*-value <2.2e-16) and with the unassigned cells (χ2 statistic:12661, df = 9, *p*-value <2.2e-16). This supports the use of cell source as the basis for categorizing blastoid models into subtypes.

We observe three clusters, the first showing a balanced cell-type composition and consisting of the blastocyst datasets and the Yanagida et al.^1^ blastoid. An enrichment of EPI_ICM cells describes the second cluster, which consists of the Yu et al.^2^ (5iLA and PXGL) and Kagawa et al.^6^ blastoid models. The third cluster consists of Liu et al., Fan et al., and Sozen et al. blastoid models,^3^^,^^4^^,^^5^ characterized by an increased abundance of PE cells and many unknown cells. Inclusion of unlabeled cells preserves Cluster 1 and Cluster 3, with Cluster 2 falling in the spectrum between the former two clusters (Figure S2F). In Figure 1C, the unknown cells in Yu et al.^2^ (both 5iLA and PXGL) and Liu et al.^3^ bridge between known cell types, suggesting intermediate states. Some peripheral cells remain in the blastocyst datasets that could present alternative lineages. These results reveal the nature of the cell-type proportions, which leads to the difference between the blastoid models and the natural blastocyst.

The cell types were assigned based on a defined set of markers. Next, we leverage the complete transcriptomic profile to determine similarities between datasets, which uncovers similarities in known cell-type identities and unknown cells in the data. We integrate the datasets to create a unified transcriptomic landscape, illustrated in Figures 1C and 1D, to evaluate the transcriptomic resemblance between blastoids and natural blastocysts. In this shared space, EPI_ICM and TE cell types are clearly distinguished, whereas PE cells split into two groups at opposite ends of the spectrum in Figure 1C. This separation indicates a transcriptomic diversity within the PE cell population across our datasets. We assign them independent labels as PE I and PE II, as indicated in Figure 1D. It appears that PE II subtype is particularly enriched for EPSC-based blastoids, but a small population could also be found in Yu2021 blastoids.

For a comparative analysis, we categorize the datasets based on their cluster distributions, as shown in Figure 1E. The groups corroborate the clustering observed in cell-type composition with some variation. The balanced cluster includes the blastocyst^1^^,^^11^^,^^13^ and the Yanagida et al.^1^ blastoid datasets. The PE-enriched blastoids, Liu et al.,^3^ Sozen et al.,^4^ and Fan et al.,^5^ vary the most with respect to their cluster distribution, with a reduced distinction for the Liu et al.^3^ blastoid than the other two models. The EPI_ICM-enriched blastoids, Kagawa et al.^6^ and the Yu et al.^2^ pair, are also distinct in their cluster distribution, although to a lesser degree than the PE-enriched blastoids. Kagawa et al.^6^ specifically shows more proximity to the balanced composition datasets than the Yu et al.^2^ dataset pair. This classification echoes the earlier cell-type abundance patterns, albeit with nuanced shifts, such as Liu et al.^3^ and Kagawa et al.^6^ moving closer to the blastocyst group.^1^^,^^11^^,^^13^ To understand the source of this variability, we observe the cluster distribution and its cell-type composition, as shown in Figure 1F. We observed that inter-group cluster distributions notably segregate. The balanced composition cluster datasets are mainly in well-defined clusters, such as clusters 5 and 3 for EPI_ICM, 33 for PE, and 29, 21, and 15 for TE. The Yu2021^2^ data pair is more abundant in clusters 6, 12, and 24 for EPI_ICM, 2 for PE, and 14 for TE. The clusters, however, include many unassigned cells. This is particularly the case for Fan et al.^5^ and Sozen et al.,^4^ with many ambiguous clusters with notable PE enrichment. There are some exceptions, such as some overlap in EPI_ICM identity between Fan et al.^5^ and Kagawa et al.^6^ in cluster 30 or EPI_ICM overlap between Kagawa et al.^6^ and Yu et al.^2^ data pair in clusters 6 and 12, indicating subtle deviances in EPI_ICM identity from blastocysts for blastoid models Kagawa et al.^6^ and Liu et al.^3^ Also, the alternative PE cluster 1 is particularly enriched for the Liu et al. blastoid dataset.

The similarities between the blastoids and blastocysts are highlighted following quantification and clustering. In Figure 1G, the similarity in distribution across all clusters reflects the same three groups reported in the overall comparison of Figure 1E. The same groups are observed with other integration methods, which reflects the biological source of the signal (Figure S3). It becomes clear, however, that the Liu et al.^3^ model occupies a peculiar position in the transcriptomic landscape, as it falls between PE-enriched blastoids Sozen et al. and Fan et al.^4^^,^^5^ and EPI_ICM-enriched blastoids Yu2021 5iLA/PXGL,^2^ even when considering only shared clusters in Figure 1H. However, more nuance is revealed in inter-group similarities in Figure 1H. High similarities between the balanced cell-type composition datasets were observed, even recovering the similarity between Yanagida et al.^1^ and the blastocysts. Whereas Kagawa et al.^6^ clusters better with other EPI_ICM-enriched blastoids (Yu2021 5iLA/PXGL).^2^

Overall, we identify three blastoid model subtypes with characteristic cell-type abundances with either balanced, EPI_ICM-enriched, or PE-enriched compositions. The three subtypes correspond to mainly blastocyst, a subset of nPSC-blastoids, and fibroblast and EPSC-blastoids. Additionally, we observe two PE subtypes, which also show biased composition between the blastoid models.

### Intermediate differentiation shapes the majority of ambiguous blastoid cell population

Although the composition of cell types in blastoids corresponds to the founding cell line, there still remain many unclassified cells, leaving the identity of a significant proportion of the cells in some models unknown. To assess the potential issue of the unknown cells, we reveal their underlying bias by introducing a lineage module score. This quantifies the differences in lineage dispositions between the blastoids and blastocysts. We first focus on phenotypes expected in the preimplantation blastocyst, namely epiblast/inner cell mass (EPI_ICM), PE, and TE cells. Here, we explore the preimplantation lineages' marker expression (Cell-Type Marker Table) across all datasets. Not all markers are expressed in all the datasets, as seen in Figure S4. Of these markers, we notice *KLF17* (EPI_ICM) is not expressed in Sozen et al.^4^ and Fan et al.^5^ We also observe the absence of *TACSTD2* (TE) in multiple datasets. *FABP3* (TE) is expressed in two separate cell groups.

**Figure 2:**
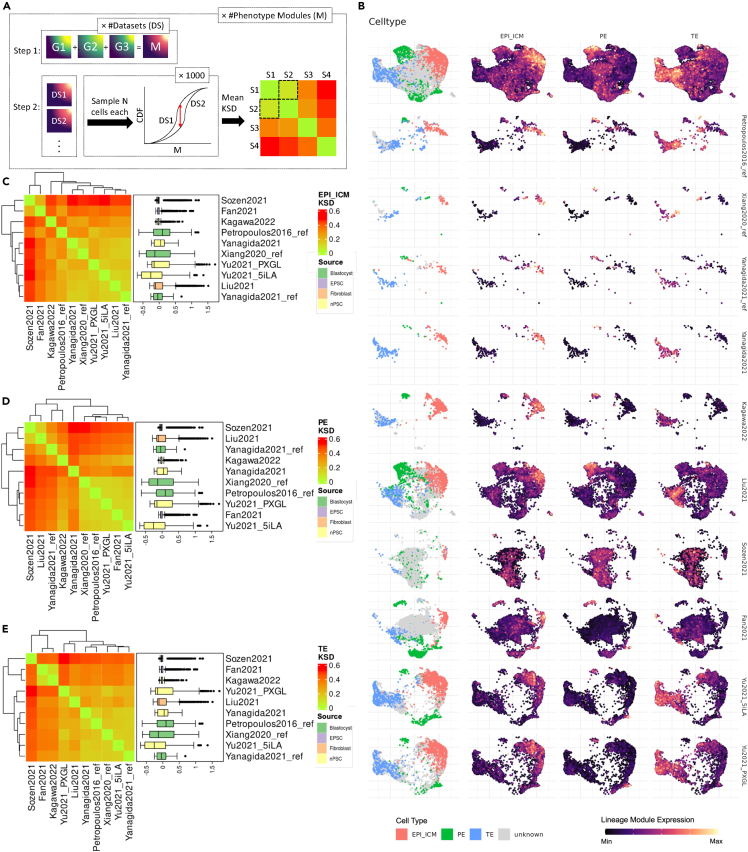
Evaluation of preimplantation lineage module scores and their distribution across datasets (A) Schematic representation detailing the calculation of phenotype module scores and Kolmogorov-Smirnov distances to assess score distribution differences. (B) UMAP visualization highlighted by the normalized expression of the cell-type labels, EPI_ICM, PE, and TE module scores, from left to right. The top figure displays all datasets within the shared transcriptomic landscape, whereas bottom figures illustrate dataset-specific labels and module score expressions in the shared landscape. (C) Heatmap depicting the KSD for the EPI_ICM score among the datasets, annotated by module score distributions. (D) Analogous to (B), focusing on the module score for the PE. (E) Similar to (B–D), detailing the analysis for the TE module score. G, marker genes; M, phenotype module; DS/S, datasets/sample; CDF, cumulative distribution function. The boxplots presented show median ±0.25 quantiles (25^th^–75^th^ percentile) with arms extending to ±0.40 quantile (10^th^–90^th^ percentile), unless specifically described.

We generate a collated module score for added lineage detection robustness in our analysis, as illustrated in Figure 2A. We first evaluate the expression of the module scores in Step 1 and observe how they are expressed in relation to their respective cell types in Figure 2B. The observed overlap is expected, given that our annotations are based on the same markers. However, our primary interest lies in the expression levels of the module in cells that are currently uncharacterized. In Petropoulos et al.,^11^ few unknown cells exist close to the trophectoderm region, which is also noticed to have higher TE module score expression. Few unknowns are observed for Xiang et al.^13^ as well. Although they are also adjacent to trophectoderm cells, they have low TE module score expression, indicating the presence of some sublineage or an emerging downstream cell type. Yanagida et al.^1^ unknown cells seem to be intermediate cells between EPI_ICM and PE, suggesting they are maturing preimplantation blastocyst cell types (Figure 2B). Most of the unknown cells in the study of Kagawa et al.^6^ are adjacent to trophectoderm cells, with a small cluster of unknown cells adjacent to the type II PE cells (Figure 2B). We observe a significant number of unknown cells for the other blastoid models. For Liu et al.,^3^ we observe intermediate cells between the two PE types, which carry some degree of EPI_ICM and PE expression (Figure 2B). Sozen et al.^4^ shows poorly defined cell types, with cell types having low phenotype scores and unknown cell types expressing all lineage module scores, preventing a precise cell identity determination. This is reinforced by the Sozen et al.^4^ blastoid’s PE and unknown cells having a distinct transcriptomic profile, as they minimally overlap with other blastocysts or blastoid cells, as we have previously observed in the last section (Figure 2B). An exception is found in Fan et al.,^5^ with some overlap in the transcriptomic profile with Sozen et al.^4^ Fan et al.,^5^ however, has a good correspondence between the cell-type labels and the expression of their respective lineage scores (Figure 2B). Although, the type II primitive endoderm shows low expression levels relative to type I. The unknown cells mostly fall between intermediate EPI_ICM and TE scores, forming a spectrum. Similarly, Yu et al.^2^ dataset pairs show good correspondence between cell-type label and score, with type I and II primitive endoderm cells present and unknown cells falling in a spectrum between EPI_ICM and TE (Figure 2B).

We further refine our analysis by quantitatively comparing the underlying score distribution between datasets. The KS distance is measured between the lineage module scores of the different datasets using a bootstrapping approach (see details in Supplementary S5). In Figure 2C, the EPI_ICM module score has a smooth variation between datasets, with most datasets having the majority of their cells with intermediate expression. Fan et al.^5^ and Sozen et al.,^4^ however, have a polar expression in their cell population, with most cells with high module score expression relative to a minority. Kagawa et al.^6^ and Petropoulos et al.^11^ show a similar pattern, although at a reduced level. Unlike EPI_ICM, the PE module score shows two alternative distribution profiles between datasets in Figure 2D. The first is with a polar expression of the lineage module. This includes Sozen et al.,^4^ Liu et al.,^3^ Yanagida et al.,^1^ and, to a lesser degree, Yanagida et al.^1^ and Kagawa et al.^6^ Alternatively, the other datasets show a spectrum of lineage module expression. For blastocysts, this could be explained by Yanagida et al.^1^ only including D6 and D7, therefore carrying more mature cells, whereas Petropoulos et al.^11^ includes D5 cells, which might still have intermediate states. As for the TE module score in Figure 2E, we observe the relative similarity between the majority of the datasets, with scores varying greatly between cells, the exception being Sozen et al.^4^ Fan et al.^5^ and Kagawa et al.^6^ However, it is more likely to be an artifact of most cells lacking the module score in many of their cells, as we see in Figure 2B for the respective datasets. This makes the TE module score unlikely to be a source of variation for the overall distribution.

Overall, the findings indicate that most unidentified cells are intermediates between EPI_ICM and TE, suggesting that these cells are either immature or still plastic, which is known in mouse ICM.^15^ We also report the PE subtypes and assess their potential as hallmarks of blastoid models.

### Amnion traits characterize primitive endoderm subtype II

Although we have characterized the preimplantation cell types across the different blastoid models in the previous sections, the question remains about the nature of PE cells in blastoid models and whether they are PE or AMN cells. Additionally, TEs are progenitors to many cell types essential for implantation, such as STBs and EVTs. In this section, we investigate their corresponding transcriptomic signals to further resolve lineage biases for the respective cell types and further interrogate the nature of unknown cell identities. Here, we use our module score technique to further disentangle the sources of transcriptional variation between different blastoids.

Similar to what was done for the primary cell lineages, we define the lineage module scores, defining the lineages observed in post-implantation blastocysts, such as AMN, EVT, and STB. First, we assess the localization of transcriptional signals for each dataset and compare them to the hotspots in the integrated landscape. In Figures S5 and S6, different phenotypes show different patterns among their markers. For AMN, the markers do not necessarily colocalize across markers or datasets. *BAMBI* and *KRT19* show notable expression but localize differently (Figure S5). Although *COL5A1* is localized with *BAMBI*, it is not highly expressed. The same phenomenon is observed for *KRT7* in EVT or *TBX3* in STB (Figures S5 and S6, respectively). Some markers are undetected in specific datasets, making a marker-based comparison unreliable. We, therefore, assess dataset similarity using phenotype module scores.

**Figure 3:**
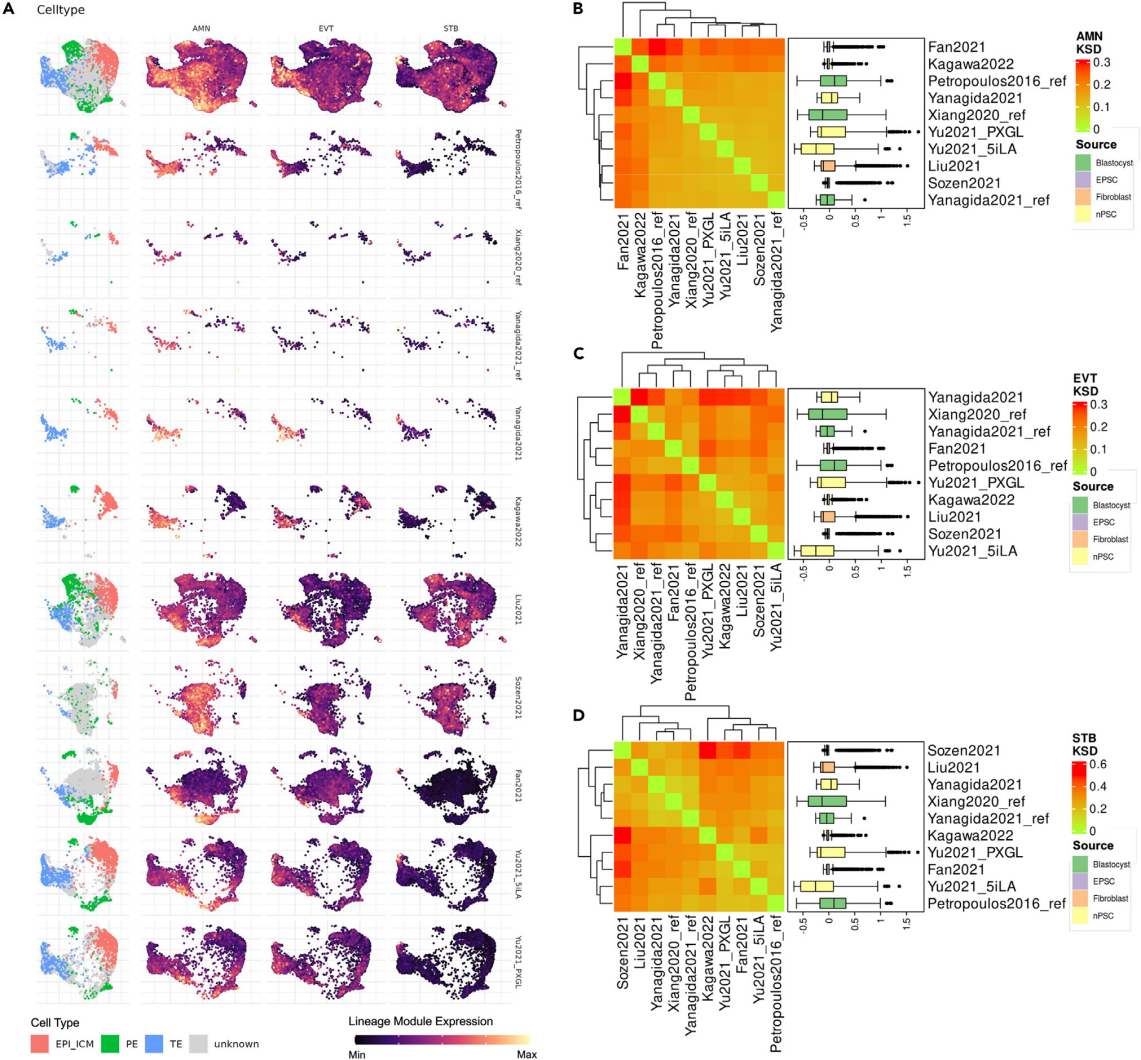
Evaluation of post-implantation lineage module scores and their distribution across datasets (A) UMAP visualization highlighted by the normalized expression of the cell-type labels, AMN, EVT, and STB module scores, from left to right. The top figure displays all datasets within the shared transcriptomic landscape, whereas bottom figures illustrate dataset-specific labels and module score expressions in the shared landscape. (B) Heatmap depicting the KS distance for the AMN score among the datasets, annotated by module score distributions. (C) Similar to (B), this set focuses on the module score for EVT. (D) Similar to (B) and (C), this time examining the STB module score. All boxplots presented show median ±0.25 quantiles (25^th^–75^th^ percentile) with arms extending to ±0.40 quantile (10^th^–90^th^ percentile), unless specifically described.

In our analysis of post-implantation markers shown in Figure 3A, we juxtapose the aggregate lineage scores with the cell-type localization in the transcriptomic landscape. The AMN phenotype correlates with intermediate cell types between the trophectoderm and type II PE. Localization in type I PE is also present but relatively restricted. Specifically, in the Liu et al.^3^ model, there’s a notable contrast between AMN module expression in type I PE and unknown cells proximal to type II PE, with the former having lower expression than the latter. In Fan et al.,^5^ type I PE cells that overlap with unknown cells from Liu et al.^3^ exhibit an elevated AMN module score. This pattern is replicated in the Yu2021^2^ dataset, where the unknown cells proximal to trophectoderm cells in the transcriptomic landscape show high levels of AMN expression, hinting at their potential identity. This transcriptional trend continues with Sozen et al.,^4^ where a high AMN lineage signal is detected across all the unknown cell populations. As for TE daughter lineage phenotypes, EVT module scores align with a subset of TE cells, indicating a possible emerging lineage bias. The STB module scores are more pronounced in expression, which, although associated with TE cells, have more restricted expression patterns. STB-phenotype-expressing TE cells are identified in all analyzed blastocysts and blastoids, except for Liu et al.^3^ and Sozen et al.^4^ This may indicate a divergence in developmental trajectory or cell differentiation potential in these models. Figure 3B shows the general similarity in overall AMN score distribution over all cells. EVT in Figure 3C shows variation between datasets, albeit no clear associations are observed. STB module expression does show clustering in Figure 3D, but a deeper look into the distributions shows little difference.

All taken together, we show that cells with AMN disposition predominantly fall between TE and type II PE, even though a subset of type I PE does show AMN module expression. We also show that a disposition toward STB and EVT lineages is observed in blastoid models, whereas weak STB and EVT dispositions are observed in blastocysts.

### Signaling pathways’ activity provide potential targets for enhancing blastoid protocols

The success of blastoid formation relies on instructing the underlying cellular programs to ensure proper cell types emerge. To determine the molecular programs across the different blastoids, we quantify the activity of various pathways targeted by blastoid culturing protocols.

**Figure 4:**
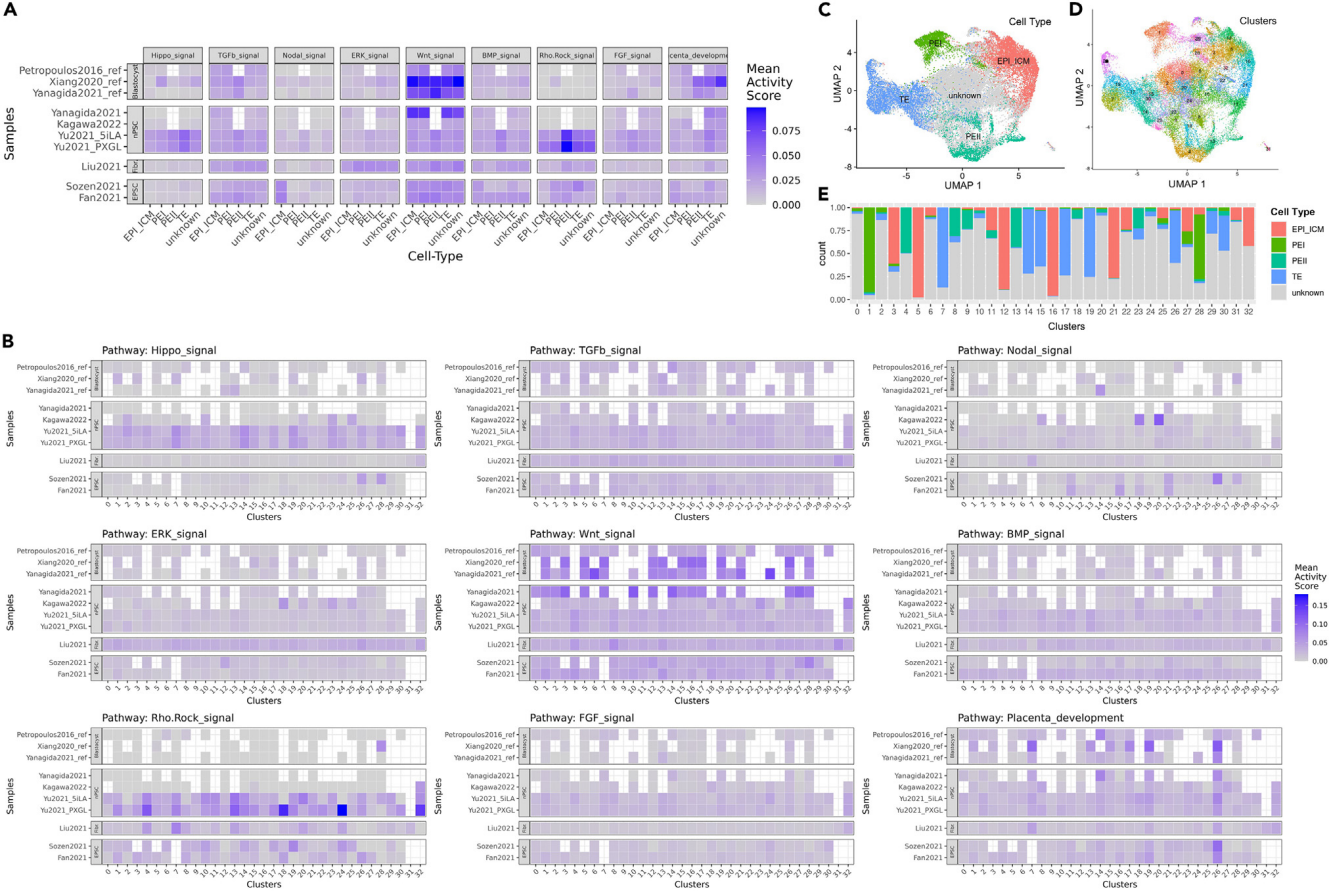
Functional pathway enrichment (A) The mean activity score (AUC score) of the pathways for each dataset with respect to the cell types. The datasets are grouped with respect to their starting cell line. White cells are empty and have no cells for the corresponding cell type. (B) The mean activity score (AUC score) of the pathways for each dataset with respect to the clusters. The datasets are grouped with respect to their starting cell line. White cells are empty and have no cells for the corresponding cluster. (C and D) UMAPs showing the cell type and clusters respectively, for reference. (E) The cell-type proportions for each clusters is shown for reference. Fibr., fibroblast.

Our results reveal pathway activity within the different datasets, as shown in Figure 4A. For example, the Yu2021 blastoid pair (5iLA and PXGL) was enriched for the Hippo-pathway-related genes. At the same time, Liu et al. fibroblast-based blastoid was enriched with ERK-signal-related genes. Cell-type-specific enrichments were also observed, such as the Nodal signaling pathway for EPI_ICM cells in EPSC-based blastoids, Sozen et al., and Fan et al. Some pathways were cell-type-specific without consideration for the dataset, such as the enrichment of placenta-development-associated genes in TE cells. At a cluster level, a more detailed description of the pathways is revealed in Figure 4B. Specifically, placental development genes are enriched for clusters 7, 14, 19, and 26—all of which are enriched with TE cells. Other TE-enriched clusters that do not have particularly enriched placental genes are 15 and 30, which are at the fringes of the TE cluster. Placental development genes were also highly enriched for the Xiang et al. dataset in TE-dominant clusters. These findings reveal further differences between the blastoid models and how the starting cell line and protocol would plausibly reflect on the cultured model.

Our results show that EPSC-based blastoids are abundant with PE subtype II cells, which are notably enriched with AMN-related genes. This could be explained by the low suppression of pathways such as the Nodal pathway, which induces PE formation.^16^ Alternatively, Yu2021 blastoid models are nPSC-based, yet they show high Hippo pathway activity, which, if inhibited, promotes TE formation.^6^^,^^17^ This could explain the relatively high EPI_ICM composition compared to natural blastocysts, but even when actively suppressed—such as for Kagawa et al.—we do not see a reduction in the EPI_ICM cell fraction, indicating necessity but not sufficiency, both of which future studies are needed to be conclusive. However, we do notice an absence of PE subtype II cells in Kagawa et al. and Yanagida et al., which calls for further investigation of the Hippo pathway’s role in the context of different starting cell lines. Overall, the analysis suggests that lower control of the signaling pathways should potentially explain the presence of off-target PE subtypes.

### Pluripotent marker heterogeneity supports robust blastoid formation

**Figure 5:**
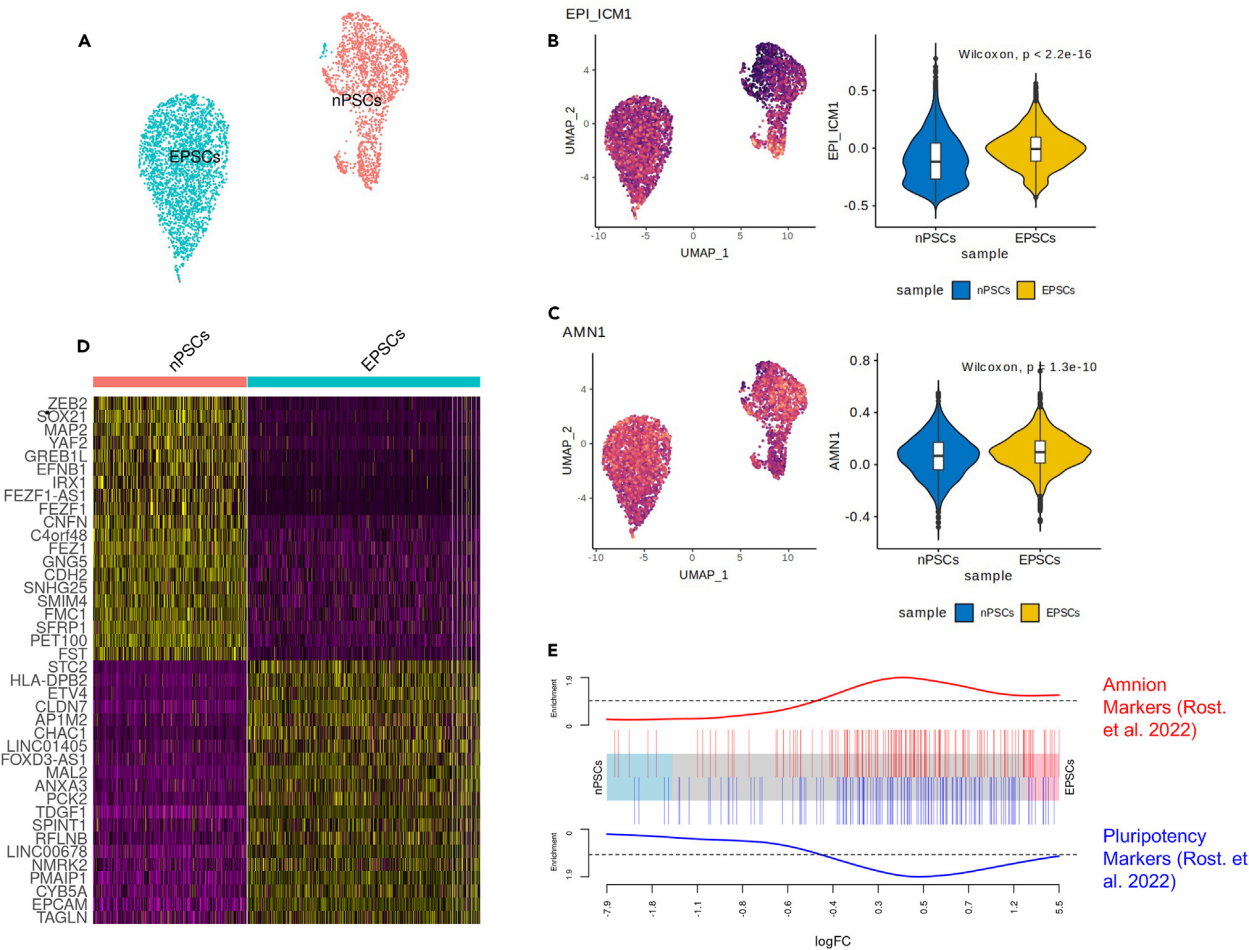
Founding cell types analysis (A) UMAP of the merged nPSCs and EPSCs scRNAseq data. (B) UMAP (right) and violin plot (left) of the EPI_ICM module score across the founding cell types. (C) Similar to (B) for AMN module score. (D) Heatmap of the expression of top 10 differentially expressed genes for each founding cell type. (E) Barcode enrichment plot for the amnion and pluripotent markers identified in Rostovskaya et al.^18^ across the top DEGs between nPSCs and EPSCs ordered by log fold change from left to right, respectively. Violin plots presented show median ±0.25 quantiles (25^th^–75^th^ percentile) with arms extending to the maximum and minimum values.

In the results of the previous sections, we observe characteristic cell-type abundance and bias to a particular lineage, such as amnionic module expression. To delineate the source of biases seen in blastoids based on EPSCs compared to PXGL nPSCs, we performed scRNAseq of EPSCs and nPSCs that were reset from the same primed iPSC line and analyzed the data (see STAR Methods section for details). The merged datasets are distinct, as seen in Figure 5A. We then investigate the biases between the two cell types. We see a polarized expression of the EPI_ICM lineage module in PXGL nPSCs (Figure 5B). Three key EPI markers, *POU5F1* (*OCT4*), *NANOG*, and *FGF4*, are more uniformly expressed in EPSCs, with *POU5F1* (*OCT4*) and *NANOG* showing elevated expression in EPSCs than in nPSCs (Figure S7). These observations are consistent with high EPI_ICM module scores of Fan et al.^5^ and Sozen et al.,^4^ which are EPSC-based blastoids.

The AMN module exhibits a more homogeneous expression in EPSCs (Figure 5C). Interestingly, canonical AMN markers *BAMBI* and *KCNMA1* are differentially expressed between ESPCs and nPSCs and have higher expression levels in more EPSC cells (Figure S7). Moreover, six (*CLDN7*, *EPCAM*, *TAGLN*, *ANXA3*, and *STC2*) out of the top twenty upregulated genes in EPSCs compared with nPSCs are AMN markers in a recent study^18^ (Figures 5D and 5E). These results suggest that the distinct AMN profile of Fan et al.^5^ (Figure 3A), which is based on EPSCs, may originate from a gene regulatory network that is more permissive to the amnionic fate already in the starting cell population.

## Discussion

This report compares blastoid models with blastocyst references, focusing on cell-type composition, distribution within the shared transcriptomic landscape, and the relevant biological signatures highlighting key lineages. By identifying the present cell types, we show characteristic cell-type compositions for blastoid model sets. We categorize blastoid models as either balanced, EPI_ICM enriched, or PE enriched. The cell-type-specific enrichment shows strong correspondence to the starting cell lines, with non-nPSC blastoids having less balanced cell-type compositions. Although, nPSC blastoids, such as Yu et al.^2^ and Kagawa et al.,^6^ show higher EPI_ICM enrichment than blastocysts. EPSC-blastoids, such as Fan et al.^5^ and Sozen et al.,^4^ are notably enriched with PE cells. This observation reinforces existing challenges toward claims of EPSC having increased totipotency potential, the ability of a cell to give rise to embryonic and extraembryonic lineages, and show that they are more restricted than conventional embryonic stem cells.^19^ Fibroblast-blastoids such as Liu et al.^3^ are also enriched with PE cells.

By establishing a joint transcriptomic landscape between all blastoids and blastocysts, we show two PE subtypes in blastoid models. While natural blastocysts and the Yanagida et al.^1^ blastoid contain only PE subtype I, other blastoids contain alternative PE subtype II. The type II PE cells were observed to be transcriptionally similar to cells with amnionic signatures. We also identify ambiguous cells that fall on a spectrum between type II PE cells and TE cells, many of which express amnionic signatures. This could explain the previously reported misclassification of amnionic cells as TE cells.^9^ In our work, we showcase the diversity of these cells and their spectrum between PE and TE, which have amnionic transcriptomic identities. There is a likelihood that the observed PE subtype II composes the encapsulating TE-like structure in the blastoids.^20^ Another interpretation of our observations would be based on previous studies that show as pluripotent stem cells (PSCs) shift from naive to primed, their lineage potential shifts from TE to AMN.^21^ The cell composition of PE and TE for the different blastoid models reflects these observations and provides an explanation for the source of these cell types. We also show subsets of TE cells expressing signatures of downstream lineages such as EVT and STB.

Alignment of the cellular identities between the different datasets unveils unique blastoid-specific signatures, such as the likely lineage profiles of the ambiguous cells and cellular subtypes. EPSC-blastoids have more ambiguous cells, indicating lower efficiency in blastoid formation. One could argue that the higher ambiguous cell composition is due to the sequencing method since 10x demands larger sample sizes, increasing the possibility of lesser-quality blastoids. However, similar abundances are observed between 10x and SMARTseq2 for non-EPSC blastoids. Most ambiguous cells fall along the EPI_ICM to TE spectrum, showing poor maturation.

To determine the impact of starting cell lines on generating observed blastoid diversity, we sequenced different founding pluripotent cell types to uncover underlying biases. A critical difference between nPSC and EPSC populations is the heterogeneity of the EPI_ICM module score. This heterogeneity implies a functional relevance in forming robust cellular compositions, a principle previously described for PSCs.^22^ Although Liu et al. utilizes this principle in their blastoid formation, our findings suggest that harnessing cellular heterogeneity as a driver for blastoid formation requires further refinement. Notably, heterogeneity has been described as a Janus-faced property: it can be conducive to blastoid formation, as observed with nPSCs, or detrimental, as seen in fibroblast-based blastoids.^23^

In addition to pluripotency marker enrichment, EPSCs show amnionic marker enrichment. If coupled with the observation of high AMN module expression in blastoids Sozen et al.^4^ and Fan et al.,^5^ it suggests a lineage bias originating from the founding cell types. The AMN markers we selected are well-recognized canonical ones. It was reported recently that the early wave of amniogenesis happens in partially primed nPSCs via a TE-like route.^18^ The AMN signatures may arise from cellular heterogeneity in the founding cell populations (e.g., EPSCs and nPSCs) that naturally harbor partially primed hPSCs.^21^

We address cross-study scRNAseq data variability using different preprocessing techniques, but it still holds that an optimal comparison would necessitate constructing the models under identical conditions. However, such an endeavor is both technically and financially prohibitive. Therefore, our analysis is a necessary step in that direction. The disparity in throughput and depth particularly impacts gene level analysis, although we reveal many signals by coarser grained analysis (i.e., cell-level, gene set modules). Also, while we highlight the role of the initial cell line in the fidelity of models, culturing conditions are undoubtedly part of optimizing these systems. While a digestion of the available protocols provides some insight to observed differences, technical limitations prevent conclusive explanations for diversity arising from protocol difference, and generating the data is not feasible for the same reasons provided before and is, therefore, beyond the scope of our work. The altering of Yanagida et al. through the excision of parts of the TE before sequencing should also be noted,^1^ although it still clustered closely to the blastocyst data.

Our work compares the blastoid models through their transcriptomic profiles. Our transcriptomic analysis provides a first blueprint of a suite of blastoid models, and a natural next step is to characterize such model using other omic modalities, such as chromatin accessibility, epigenomic modifications, and proteomic profile. Exploring the multi-omics of blastoid development is poised to reveal a deeper characterization and understanding of the origin of heterogeneity in these models. Additionally, investigating the mechanisms that lead to the formation of alternative PE subtypes is also a venue for future work. Determining the source could unveil potential targets to enhance blastoid formation efficiency further.

In conclusion, our findings highlight the cell-type distribution biases imposed by specific protocols and initial cell sources. We provide the first direct side-by-side comparison of cell line transcriptomic elements that influence the blastoid model development quality. We show that heterogeneity in canonical pluripotency markers could explain the capacity of the cell line to give rise to full blastoids. Finally, we hope our findings pave the way for the future development of early human development models.

### Limitation of the study

Although our study offers a thorough analytical comparison of different blastoid models, several limitations remain. First, the optimal scenario would involve using scRNAseq data from natural human blastocysts generated with 10x Genomics technology. Such data would allow for a more reliable comparison with 10x-sequenced blastoid datasets. However, publicly available data are of insufficient quality for reliable comparisons, and the 10x technology is highly sample-demanding, making it difficult to obtain adequate sample size of natural human blastocysts. Second, differential expression analysis across sequencing platforms presents challenges, complicating the reliable reporting of differentially expressed genes (DEGs). To mitigate these limitations, future work could reconstruct the blastoids and perform sequencing using a consistent platform, such as SMARTseq2. Third, we modeled the starting cell lines (naive PSCs and EPSCs) using iPSCs cultured under different conditions. A more robust approach might include a comparison of naive hESCs and EPSCs derived from hESCs, which could enhance and confirm our findings. Additionally, several blastoid models have been published since the submission of this manuscript, but due to time and space constraints, they were not included in our analysis. The framework we present could be applied to these new models in future studies. Finally, although our computational analysis provides valuable insights into blastoid characteristics, experimental validation is necessary to for our findings to be conclusive.

## Resource availability

### Lead contact

Requests for further information and resources should be directed to and will be fulfilled by the lead contact, Ali Balubaid (ali.balubaid@kaust.edu.sa).

### Materials availability

This study did not generate new unique reagents.

### Data and code availability

• Data: this paper analyzes existing, publicly available data, accessible at Gene Expression Omnibus (GEO) through accession numbers reported in dataset summary table and links in the key resources table. The starting cell lines scRNAseq data generated in this paper is deposited at GEO: GSE247758 accession number and are publicly available as of the date of publication.
• Code: all original code has been deposited at GitHub: https://github.com/balubao/Blastoid_scRNAseq_Comparison and is publicly available.
• Additional information: any additional information required to reanalyze the data reported in this paper is available from the lead contact upon request.

## Acknowledgments

We also thank Asima Zia, Vincenzo Lagani, Robert Lehmann, Mahdi Alshoyokh, and Kenaz Abuzenada for the fruitful discussions, as well as Alberto Maillo for his support with setting up the online resource. We acknowledge the KAUST Baseline Awards, with D.G.-C. supported by KAUST Baseline Award no. BAS/1/1093-01-01, M.L. supported by KAUST Baseline Award no. BAS/1/1080-01-01, and J.T. supported by KAUST Baseline Award no. BAS/1/1078-01-01. Graphical abstract and schematics were created with BioRender.com.

## Author contributions

Conceptualization ideas, J.T., M.L., and D.G.C.; methodology, A.B., S.A., D.G.C., and N.A.K.; software, A.B.; formal analysis, A.B.; investigation, A.B., S.A., and M.L.; resources, M.L. and J.T.; data curation, A.B. and S.A.; writing—original draft, A.B., S.A., M.L., and J.T.; writing—review & editing, A.B., S.A., N.A.K., D.G.C., M.L., and J.T.; visualization, A.B.; supervision, M.L. and J.T.; project administration, A.B.; funding acquisition, D.G.C., M.L., and J.T.

## Declaration of interests

The authors declare no competing interests.

## Declaration of generative AI and AI-assisted technologies in the writing process

During the preparation of this work, the author(s) used chatGPT to improve readability and flow. After using this tool/service, the author(s) reviewed and edited the content as needed and take(s) full responsibility for the content of the published article.

## STAR★Methods

### Key resources table

**Table:** 

REAGENT or RESOURCE	SOURCE	IDENTIFIER
**Biological samples**		

iMEF	In-house	NA
nPSCs	In-house	NA
EPSCs	In-house	NA

**Chemicals, peptides, and recombinant proteins**		

DMEM/F12	Thermo Scientific	11330032
Neurobasal	Thermo Scientific	21103049
N2	Thermo Scientific	17502–048
B27	Thermo Scientific	12587010
PD0325901	VWR	101761–458
LIF	Cell Signaling	62226S
VPA	SIGMA	P4543-10G
XAV-939	VWR	ALEXBML-WN100-0005
Gö6983	VWR	BIOV9539-5
CHIR99021	SIGMA	SML1046-5MG
(S)-(+)-Dimethindene maleate	ZZZ-UTECH	5365393
Minocycline hydrochloride	SIGMA	M9511-100MG
IW-1	SIGMA	I0161-5MG
Y-27632	VWR	101763–968

**Critical commercial assays**		

10X Chromium Single Cell 3′ Solution V2 Reagent Kits	10x Genomics	https://www.10xgenomics.com/products/single-cell-gene-expression

**Deposited data**		

Human reference genome GRCh38	Ensembl Genome Browser	https://asia.ensembl.org/Homo_sapiens/Info/Index
Starting cell lines (EPSCs/nPSCs)	This paper	https://www.ncbi.nlm.nih.gov/geo/query/acc.cgi?acc=GSE247758
Petropoulos et al.	Petropoulos et al.^11^	https://www.ebi.ac.uk/biostudies/arrayexpress/studies/E-MTAB-3929/; E-MTAB-3929
Xiang et al.	Xiang et al.^13^	http://www.ncbi.nlm.nih.gov/geo/query/acc.cgi?acc=GSE136447; GSE136447
Yanagida et al.	Yanagida et al.^1^	https://www.ncbi.nlm.nih.gov/geo/query/acc.cgi?acc=GSE171820; GSE171820
Yanagida et al.	Yanagida et al.^1^	https://www.ncbi.nlm.nih.gov/geo/query/acc.cgi?acc=GSE171820; GSE171820
Kagawa et al.	Kagawa et al.^6^	https://www.ncbi.nlm.nih.gov/geo/query/acc.cgi?acc=GSE177689; GSE177689
Liu et al.	Liu et al.^3^	https://www.ncbi.nlm.nih.gov/geo/query/acc.cgi?acc=GSE156596; GSE156596
Sozen et al.	Sozen et al.^4^	https://www.ncbi.nlm.nih.gov/geo/query/acc.cgi?acc=GSE178326; GSE178326
Fan et al.	Fan et al.^5^	https://www.ncbi.nlm.nih.gov/geo/query/acc.cgi?acc=GSE158971; GSE158971
Yu et al.	Yu et al.^2^	https://www.ncbi.nlm.nih.gov/geo/query/acc.cgi?acc=GSE150578; GSE150578
Yu et al.	Yu et al.^2^	https://www.ncbi.nlm.nih.gov/geo/query/acc.cgi?acc=GSE150578; GSE150578

**Software and algorithms**		

Sratoolkit (v2.11.0)	GitHub	https://github.com/ncbi/sra-tools/wiki/01.-Downloading-SRA-Toolkit
FastQC (0.11.8)	Babraham Bioinformatics	https://www.bioinformatics.babraham.ac.uk/projects/download.html#fastqc
Trimmomatic (v0.38)	Bolger et al.^24^	https://github.com/timflutre/trimmomatic
Kallisto (v0.44.0)	Bray et al.^25^	https://pachterlab.github.io/kallisto/
Cell Ranger (v6.1.2)	Zheng et al.^26^	https://support.10xgenomics.com/single-cell-gene-expression/software/overview/welcome
R (v4.4.1)	R Core Team (2017). R: A language and environment for statistical computing. R Foundation for Statistical Computing, Vienna, Austria.^27^	http://www.r-project.org/; RRID:SCR_001905
Seurat (v5.0.1)	Hao et al.^28^	https://satijalab.org/seurat/
SCINA (v1.2.0)	Zhang et al.^29^	https://github.com/jcao89757/SCINA
SCType	Ianevski et al.^30^	https://github.com/IanevskiAleksandr/sc-type
SingleR (v2.6.0)	Aran et al.^31^	https://www.bioconductor.org/packages/release/bioc/html/SingleR.html
Seurat-Disk	GitHub^32^	https://github.com/mojaveazure/seurat-disk
Seurat-Wrapper	GitHub	https://github.com/satijalab/seurat-wrappers
Harmony (v1.2.1)	Korsunsky et al.^33^	https://github.com/immunogenomics/harmony
Stats	R Core Team (2017). R: A language and environment for statistical computing. R Foundation for Statistical Computing, Vienna, Austria.^27^	https://www.r-project.org/
philentropy (v0.8.0)	Drost et al.^34^	https://cran.r-project.org/web/packages/philentropy/
Pheatmap (v1.0.12)	Kolde^35^	https://cran.r-project.org/web/packages/pheatmap/
AUCell (v1.26.0)	Aibar et al.^36^	https://www.bioconductor.org/packages/release/bioc/html/AUCell.html
Code	This paper	https://github.com/balubao/Blastoid_scRNAseq_Comparison

### Experimental model and study participant details

#### Founding cell line culture

To investigate the lineage biases in the founding cell lines, we culture and sequence nPSC and EPSC populations. Induced pluripotent stem cells (iPSCs) used in this study termed SC-9N is a female line used in numerous studies.^37^^,^^38^^,^^39^^,^^40^ This cell line was converted to nPSCs and EPSCs following published protocols.^41^^,^^42^ Briefly, for nPSCs, primed iPSCs were chemically reset on iMEF using N2B27 as basal media supplemented with 1 *μ*M PD0325901 (Selleck, S1036), 10 ng/mL LIF (R&D Systems, 7734), and 1 mM VPA (Sigma, PHR1061) for three days. This was followed by propagating these cells in PXGL media, which consists of 1 *μ*M PD0325901 (Selleck, S1036), 2 *μ*M XAV-939 (Selleck, S1180), 2 *μ*M Gö6983 (Axon, 2466), and 10 ng/mL LIF (R&D Systems, 7734). Primed PSCs were treated with an EPS medium termed LCDM, using N2B27 as a basal medium to generate EPS. LCDM medium was comprised of N2B27 basal medium supplemented with 10 ng/mL human LIF (R&D Systems, 7734), 3 *μ*M CHIR99021 (Tocris, 4423), 2 *μ*M (S)-(+)-Dimethindene maleate (Tocris, 1425), and 2 *μ*M minocycline hydrochloride (Selleckchem, S4226), 1 *μ*M IWR endo-1 (Selleckchem, S7086), and 2 *μ*M Y-27632 (Selleckchem, S1049). Both nPSCs and EPS were passaged until domed-shaped colonies appeared in the culture before being used for scRNAseq. Naive PSCs were cultured in conditions of 5% CO₂ and 5% O₂, while EPSCs were maintained in 5% CO₂ and 21% O₂.

### Method details

#### Public blastoid and blastocyst datasets

We collected all blastoid models with available scRNAseq data. Of the six published blastoid models, those by Yu et al.^2^ included two successful models of nPSCs cultured in either 5iLA or PXGL medium.^2^ Each model was evaluated separately. We used three reference datasets in our analysis as controls. Our references include datasets describing the natural blastocyst within the E5 to E7 time window, published in two datasets spanning days 5–7 and 5–6, respectively.^1^^,^^11^ Another reference we use is that of an *in vitro* cultured blastocyst grown to the primitive streak anlage stage, from which days E6-E7 capture the blastocyst stage.^13^ Our evaluation, therefore, consists of three reference datasets and seven blastoid models, totaling ten datasets. The datasets were acquired from public repositories (GSE numbers listed in dataset summary table).

**Table:** Dataset Summary

#	Dataset	Ncells (postQC)	Sequencing Platform	Ref	Accession	Description
1	Petropoulos et al.^11^	1232	SMARTseq2	✓	E-MTAB-3929	Early human blastulation (E5-E7)
2	Xiang et al.^13^	127	SMARTseq2	✓	GSE136447	Human Development *ex vivo* (E6-E7)
3	Sozen et al.^4^	4918	10x v3	–	GSE178326	EPSC blastoid model (D5/6)
4	Liu et al.^3^	7836	10x v3	–	GSE156596	Reprogrammed Fibroblasts (Day 6)
5	Yu et al.^2^	5028	10x v3	–	GSE150578	nPSC; HT; PXGL (Day 9)
6	Yu et al.^2^	4458	10x v3	–	GSE150578	nPSC; HT; 5iLA (Day 9)
7	Yanagida et al.^1^	267	SMARTseq2	–	GSE171820	nPSC
8	Yanagida et al.^1^	228	SMARTseq2	✓	GSE171820	Early Human Blastocyst (E6)
9	Fan et al.^5^	10879	10x v3	–	GSE158971	EPSC (Day 6)
10	Kagawa et al.^6^	1210	SMARTseq2	–	GSE177689	nPSC with tri-inhibition (96h)

#### Mapping to a shared genome reference

To eliminate bias due to gene labeling and genome reference versions, all datasets were remapped to the human genome reference GRCh38 (v105).^43^ For 10x datasets, we used Illumina’s Cell Ranger pipeline (v6.1.2)^26^ with default parameters. For SMARTseq2 datasets, raw fastq files were quality checked,^44^ and trimmed using Trimmomatics (v0.38)^24^ with the following parameters: LEADING:3, TRAILING:3, SLIDINGWINDOW:4:15, MINLEN:32, and ILLUMINACLIP set to NexteraPE-PE.fa:2:30:10 adapters. Reads were then mapped using Kallisto (v0.44.0)^25^ with default parameters, adjusting to `-l 200 –single` for datasets with single-end reads. Transcript per million (TPM)^45^ estimates were generated for SMARTseq2 data, and features expressed in less than 20 cells were filtered out.

#### Count matrix assembly

For SMARTseq2 datasets, TPM matrices were assembled, with columns as cells and rows as features (transcripts identified by Ensembl transcript IDs). All transcript isoforms of a gene were treated as a single feature by summing the counts of transcripts corresponding to the same Ensembl gene ID, and then substitute with gene symbols if available. For 10x data, we loaded the filtered count matrix and created a Seurat (v5.0.1)^28^ object. For both platforms, features found in fewer than 10 cells were filtered out, and cells with fewer than 100 features were also excluded to reduce noise and enhance computability.

#### Filtering outlier cells

Outlier cells were identified based on the number of features or counts, with thresholds set using density inflections, similar to the Cell Ranger knee inflection method for identifying empty droplets. The number of counts filter was only applied to 10x data, since SMARTseq2 uses TPMs that normalize counts to a sum of approximately 1 million per cell. For mitochondrial read ratios, we set a filter threshold at 20%, with adjustments based on the mitochondrial read density for each dataset. Thresholds are summarized in Table S1 - QC&PP.

#### Normalization and scaling

All datasets were normalized using pseudo-log transformation with the ‘logNormalization’ function, The top 3000 variable features were then identified using variance stabilized transformation (vst) with the ‘FindVariableFeatures’ function. The data was then scaled with ‘ScaleData’ while regressing out spike-in read percentage (ERCC.perc) when available (i.e., Petropoulos et al.), number of features (nFeatures), number of counts (nCounts), and cell-cycle effects (S.Score and G2M.Score). These adjustments were made to remove technical biases such as PCR amplification, feature abundance, and cell-cycle phase influences. For downstream analysis, we scale the top variable features and other features of interest indicated in Cell-Type Marker Table. All functions listed in this section are from the Seurat package (v5.0.1).^28^

#### Dimensionality reduction and clustering

Next, we performed principal component analysis (PCA) using the `RunPCA` function, selecting 10–15 principal components based on the `ElbowPlot` to maximize information retention. We computed cell neighbors using `FindNeighbors` and clustered the datasets using `FindClusters` with a resolution of 2.0 to ensure detection of less abundant cell types (e.g., PE) in smaller datasets like Yanagida et al. and Xiang et al. This clustering facilitated downstream anchor-based integration for subsequent analysis.^46^ All functions listed in this section are from the Seurat package (v5.0.1).^28^

#### Cell-type annotation and module computation

The cell-type markers used were acquired from literature, as detailed in Cell-Type Marker Table. We select the transcription factors that are differentially expressed in the annotated cells during the early stages, as reported by Xiang et al.^13^ and Zhou et al.^13^^,^^47^ Determining their cell type composition is the first step in comparing various blastoid models. This process necessitates the establishment of a unified annotation framework. We use marker-based (i.e., SCINA, SCType) and reference-based (i.e., SingleR) annotation methods to achieve this.^29^^,^^30^^,^^31^ We first annotate with the marker-based methods using our curated markers from Cell-Type Marker Table. We verify our annotations by mapping between the annotations inferred by SCINA^29^ and SCType^30^ with published annotations of the reference datasets.^1^^,^^11^^,^^13^ The cell types of our annotation and the published annotations align (Figures S8A and S8C). For our second annotation approach, we employ reference-based methods. We implement cell-level SingleR^31^ annotation tool on the blastoid datasets using the labels generated by cluster-level SCType.^30^ This allows us to harmonize the labels between the different references, providing a shared basis for downstream analysis. Given these annotations from the different methods, we evaluate our results. First, SCINA^29^ assigns more 'unknown' labels than SCType,^30^ showing its high specificity (Figure S8D). SingleR^31^ also shows notably higher sensitivity, and the majority of cells are assigned (Figure S8D). For robust label assignment, we take a majority vote for each cell between cluster-level SCType, SCINA, and cell-level SingleR (Figures S2A–S2D and S9E). The cell-type lineage profile for each cell was estimated using lineage module scores computed from the average expression of selected set lineage-specific markers normalized against random genes expressed within the same magnitude using Seurat’s `AddModuleScore`, based on the method of Tirosh et al..^48^

**Table:** Literature Procured Cell-Type Marker Genes^13^^,^^47^

Cell Type	Positive Markers	Symbol
Epiblast/Inner cell mass	SUSD2, NANOG, KLF17, FGF4, BMP2, LAMA4	EPI_ICM
Primitive endoderm	GATA4, PDGFRA, SOX17, GATA6	PE
Trophoblast cells	GATA3, TACSTD2, FABP3	TE
Amnion cells	BAMBI, COL5A1, ISL1, KCNMA1, KRT19, PODXL, VTCN1	AMN
Extravillous cytotrophoblasts	CSH1, CSH2, DLX5, DLX6, ERBB2, HLA-G, KRT7, MMP2, RXRA	EVT
Syncytiotrophoblasts	CGB1, CGB2, CGB5, CGB7, CGB8, ERVFRD-1, ESRRG, FOXO6, MAFK, PSG3, TBX3, TCL6	STB

#### Integration of the datasets

scRNAseq dataset integration is used to correct high sources of technical variation, such as batch effects and sequencing platform differences. Such a correction allows for a more effective comparison of cellular composition and distributions between different datasets. For the integration-based analysis in this study, we select an integration method that makes the least number of assumptions possible on the data with a more bottom-up approach to integration. Our rationale is that we expect the different blastoid models to capture essential elements of the human blastocyst, but we also expect some biological uniqueness due to the distinct culturing protocols. To that end, we integrate our data using ` IntegrateData` from the `Seurat` (5.0.1)^28^ package, applying the reciprocal principal component analysis (RPCA) method without a reference using ‘method = RPCAIntegration’ and defining `k.weight = 80′ to accommodate for all dataset sizes. Other integration methods were also tested, such as canonical component analysis (CCA) and mutual nearest neighbor (MNN),^49^ by defining ‘method = CCAIntegration’ and ‘method = FastMNNIntegration’, and Harmony^33^ respectively. For integrations using a reference, we specify Petropoulos et al.,^11^ Yanaigdaet al.^1^, and Xiang et al.^13^ as references. Once the data is integrated, we compute the neighbors and cluster the cells using ‘FindNeighbors’ and ‘FindClusters’ functions. The integrated data was clustered with `resolution = 2.0` as done during the preprocessing step.

#### Cell type refinement

After noting the two PE populations, we refine the definition by manual assignment using the RPCA clusters. The PEs in clusters 7, 30, 24, 11, 14, 23, 8, 9, 18, 13, 4, and 31 are defined as PE subtype II. Otherwise, the PE is considered subtype I.

#### Qualitative lineage module score analysis

For the lineage module scores, the qualitative approach visualizes the min-max normalized (values span between 0 and 1) module score for marker enrichment, where peaks can be identified in the visualized manifold and contrasted by juxtaposing the shared embedding UMAP^50^ between the datasets.

#### Pathways and processes activity analysis

We select a collection of pathways targeted by the culture protocols indicated in Figure S1, as well as physiological relevant processes. We list the pathways explored in Pathway and Processes Table, including the GO IDs used to retrieve the gene set under the term. Each dataset was enriched for the unique gene set using AUCell function,^36^ which ranks the genes by their expression and computes the area under the curve (AUC) of recovery for genes defined along their rank. We use the AUC continuous score as a measure of pathway activity.

**Table:** Pathways and Processes of Functional Relevance in Blastoids

Pathway	GO ID	Description
Hippo signaling pathway genes	0035329	Hippo inhibition promotes TE cells.^6^^,^^17^
TGF β signaling pathway genes	0007179	Maintains pluripotency (ICM), inhibition promotes TE.^6^
Nodal signaling pathway genes	0038092	Induces PE formation.^16^
ERK signaling pathway genes	0070371	Erk inhibits PE formation and promotes EPI identity.^51^ Inhibition also promotes TE formation.^6^
Wnt signaling pathway genes	0030111	Maintains EPI cells.^21^
BMP signaling pathway genes	0030509	Promotes TE.^2^
Rho/Rock signaling pathway genes	0072518	Inhibition promotes cell viability.^52^
FGF signaling pathway genes	0008543	FGF4 promotes PE at the cost of EPI formation^51^
Placenta Development genes	0001892	Formed by TE lineage progeny.

#### Founding cell line sequencing

For 10x genomics scRNAseq library preparation, 10X Chromium Single Cell 3′ Solution V2 was used; 5000 cells per cell type were targeted, single-indexed, and sequenced using the Novaseq 6000 sequencing system; both samples were run on an S1 flow cell.

#### Founding cell type analysis

The count matrices for the data were generated using the Cell Ranger pipeline (v6.1.2)^26^ from fastq read files. The cell line data was then analyzed following the standard Seurat (v5.0.1)^28^ preprocessing pipeline. The mitochondrial and ribosomal genes were removed for DEG analysis. The DEGs between source lines were identified using the ‘FindAllMarkers’ function in Seurat. DEGs were computed using the Wilcox rank-sum test and the heat maps generated by Seurat “DoHeatmap” function.

### Quantification and statistical analysis

All statistical tests and parameters used are indicated in figure legends. Sample sizes are reported in Table S1. All boxplots presented show median ±0.25 quantiles (25^th^-75^th^ percentile) with arms extending to ±0.40 quantile (10^th^-90^th^ percentile), unless specifically described. All violin plots presented show median ±0.25 quantiles (25th-75th percentile) with arms extending to the maximum and minimum values. We expand more on cell-type abundance analysis, cluster distribution analysis, quantitative lineage module score analysis tests for clarity.

#### Cell type abundance analysis

To evaluate the significance of association between source cell line (*n* = 3) and observed proportions of cell types (*n* = 3 + 1 for unknown cell types), the chi-squared test (χ^2^-test) was used to compare whether an association between the two categorical variables (i.e., source cell line versus cell type) was statistically significant. The test was done for both with and without the non-annotated cells (w. unknown OR w/o unknown) using a contingency table, and the results were reported in the text. The exact number of cell types can be found in Table S1.

#### Cluster distribution analysis

Blastoid similarities were evaluated by comparing their cluster distributions in the integrated transcriptomic landscape. The proportions are acquired by dividing the number of cells in a cluster by the sum of all cells for each dataset. We run a principal component analysis (PCA) on the cluster distributions using base `prcomp` function from the ‘stat’ package.^27^ Cluster distribution similarity was quantified using Jensen-Shannon distance (JSD) and Pearson Correlation distance (PCD). JSD measures the relative entropy between dataset distributions, it quantifies similarity using all clusters, including those not occupied by any dataset or only one. JSD is computed using the `distance` function from the `philentropy` (v0.8.0)^34^ package with the parameter `method = jensen-shannon` for the cluster proportion vectors. Whereas PCD is computed by first using the standard ‘cor’ function from the ‘stat’ package^27^ with parameter ‘use = pairwise.complete.obs’, restricting correlation computation to only clusters shared between a dataset pair. The correlation values are then translated to distances between 0 and 1 using equation:

$$PCD=\left(1-cor\left(C_{i},C_{j}\right)\right)/2$$

Where C is the non-zero cell proportion vector for datasets i and j. The distribution similarities are visualized using clustered heatmaps from the ‘pheatmap’ package (v1.0.12).^35^

#### Quantitative lineage module score analysis

To quantify the similarity in lineage module expression score, the module scores were scaled separately by the mean and variance for each score in each dataset. The Kolmogorov-Smirnov (KS) distance was then computed between the different datasets using the statistic generated by the ‘ks.test’ function from ‘stat’ package.^27^ The KS test measures the largest distance between the cumulative distribution function of two continuous vectors. In our case, a vector corresponds to the lineage module scores for all cells in a dataset. To control for different numbers of cells across the datasets, we bootstrap by subsampling each dataset using the size of the smallest dataset (Xiang et al.^13^; *n* = 122) for a 1000 iterations, and take the element-wise average to estimate the lineage score KS distances between the datasets accurately. The robustness of the results was verified with a sensitivity analysis (Figure S9).

#### KS distance sensitivity analysis

The robustness of the KS distance measure was assessed by subsampling cells at different rates (*n* = 100, 400, 800, 1000, 1500) with replacement. Figure S9 shows that while increasing subsampling size might decrease distance values, the rankings between the distances are preserved. In Figure S9A, no apparent effects of different sampling sizes are observed. A sample size-based shift is observed when computing the difference between the KS distances of each sampling size and the size used in the main text (*n* = 122) in Figure S9B. Overall, the observation is replicated when looking at the norm of the differences in Figure S9C, which indicates general deviance as the sample size increases. The AMN lineage module, mainly, shows notable shifts. However, we have mainly relied on the relative distances to understand the relationships of the module scores between datasets and, therefore, are more concerned with preserving the ranks rather than the absolute values. When looking at the rank correlations in Figure S9D, we observe an overall stability in the ranking. To further demonstrate the stability of KS distances for different sample sizes, we show the KS distance matrix for the AMN module score across datasets for multiple subsample sizes (Figure S9E). In concordance with our overall analysis, we see subtle differences if any, with generally higher contrast in distances with higher sample sizes. Overall, we show the stability of our bootstrap computation for KS distance for the lineage-specific module scores.

### Additional resources

An atlas for the different datasets analyzed in this work could be accessed through the following link: https://translationalbio.shinyapps.io/BlastoidAtlas/.

## Supplemental information

Document S1. Figures S1–S9

Table S1. Excel file with dataset details including (Sheet 1: General) general statistics and accession IDs, and (Sheet 2: QC&PP) details for QC thresholds, cell-type assignments, and lineage module score quality, related to STAR Methods
