# Supplementary material for: A comparative analysis of blastoid models through single-cell transcriptomics
Source: iScience. 2024 Oct 11;27(11):111122. doi: 10.1016/j.isci.2024.111122 (PMC11543915; doi:10.1016/j.isci.2024.111122)
Supplement: Document S1. Figures S1–S9 [file mmc1.pdf]

iScience, Volume *27*

Supplemental information

A comparative analysis of blastoid models through single-cell transcriptomics

Ali Balubaid, Samhan Alsolami, Narsis A. Kiani, David Gomez-Cabrero, Mo Li, and Jesper Tegner

Supplementary Information

SUPPLEMENTARY FIGURES

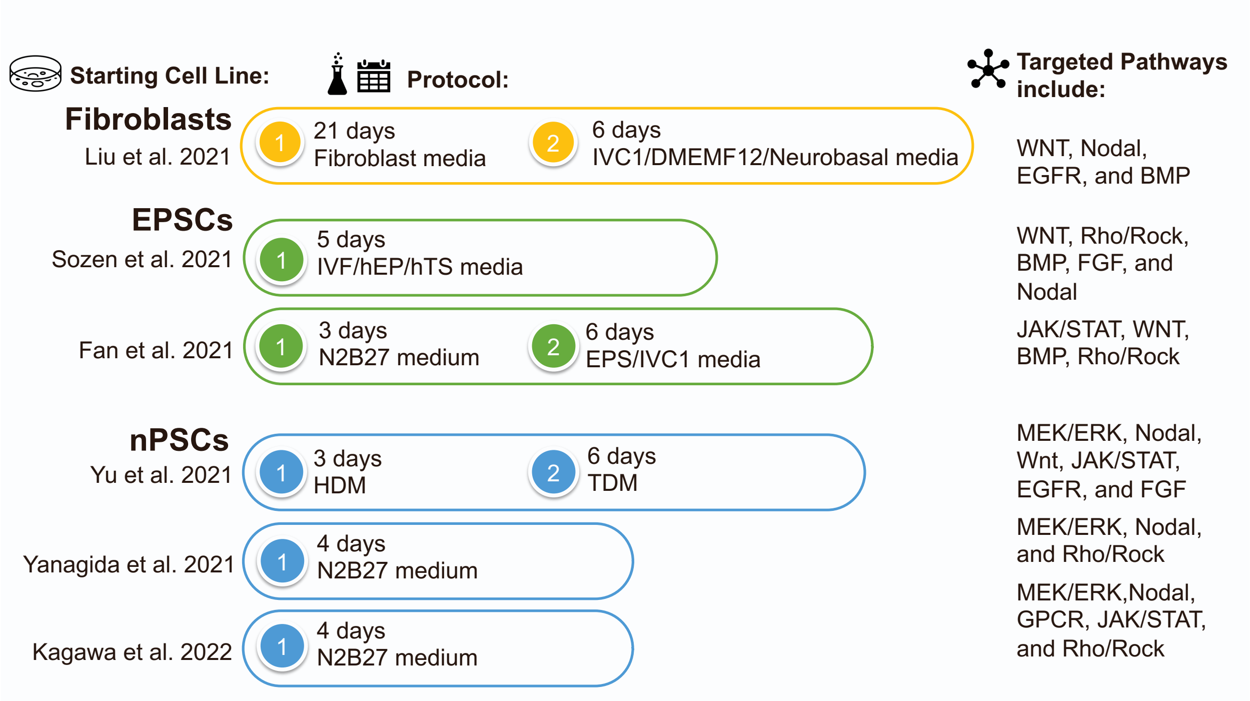

Figure S1. Blastoid Culture Protocol Schematic, Related to Introduction: A. This panel displays steps, used media, and some of the targeted pathways to make the human blastoid-like model from fibroblasts, EPSCs, and nPSCs. IVC1 stands for In Vitro Culture medium, IVF stands for In Vitro Fertilization medium, hEP stands for human Extended Potential medium, hTS stands for human Trophoblast Stem cell medium, HDM stands for Hypoblast Differentiation Medium, and TDM stands for Trophoblast Differentiation Medium.

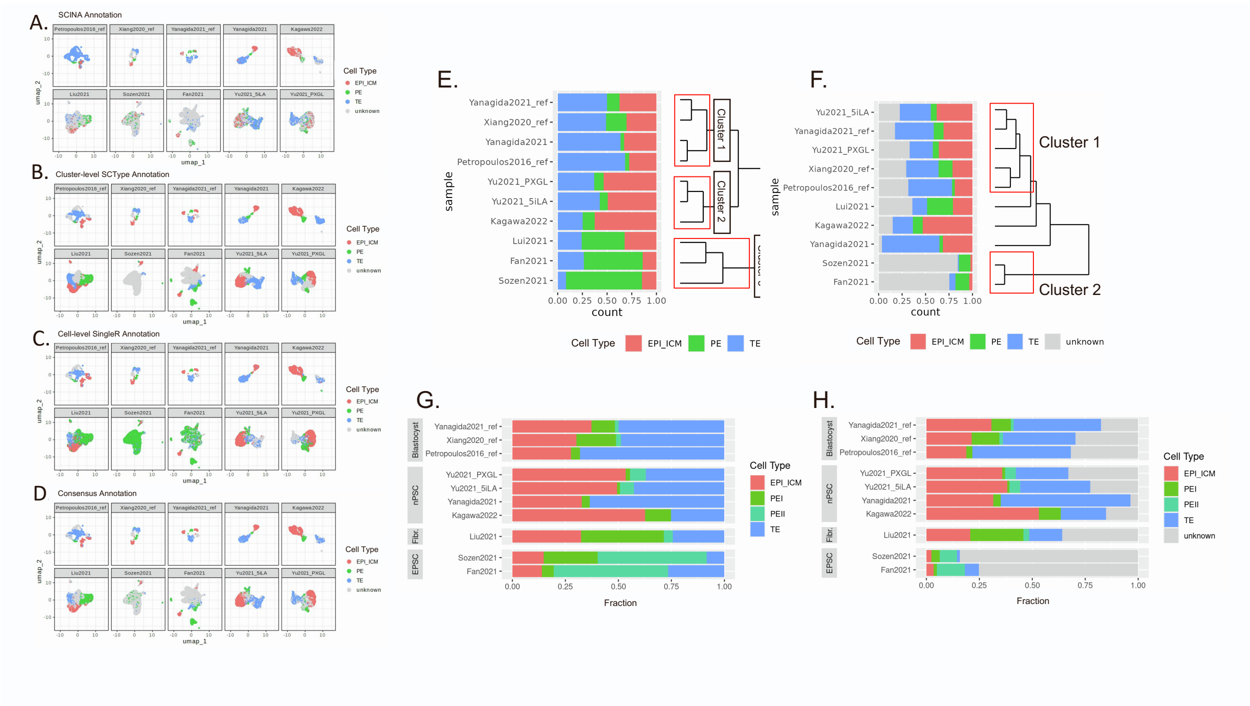

Figure S2. Cell Type Annotation UMAPs and Clustering, Related to Figure 1: UMAPs for each dataset annotated by the cell type labels for (A) SCINA, (B) cluster-level SCType, (C) cell-level SingleR, and (D) the majority vote annotation. E. Datasets are clustered using the distribution of majority annotation cell type labels, including unassigned cells.

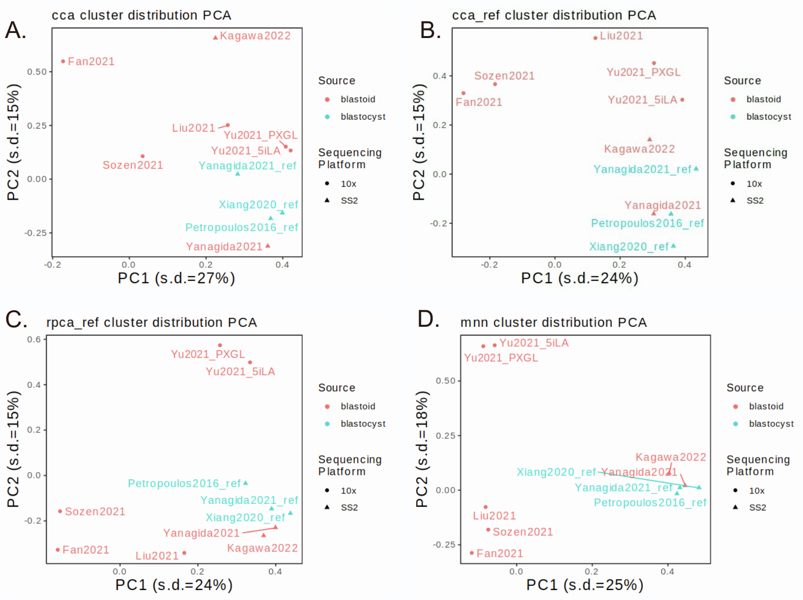

Figure S3. Cluster Distribution Embeddings for Different Integration Methods, Related to Figure 1: A. The PCA of dataset distribution across louvain clusters for cells embedded in the integrated transcriptomic landscape by (A) CCA, (B) CCA with a defined reference, (C) RPCA with a defined reference, and (D) MNN.

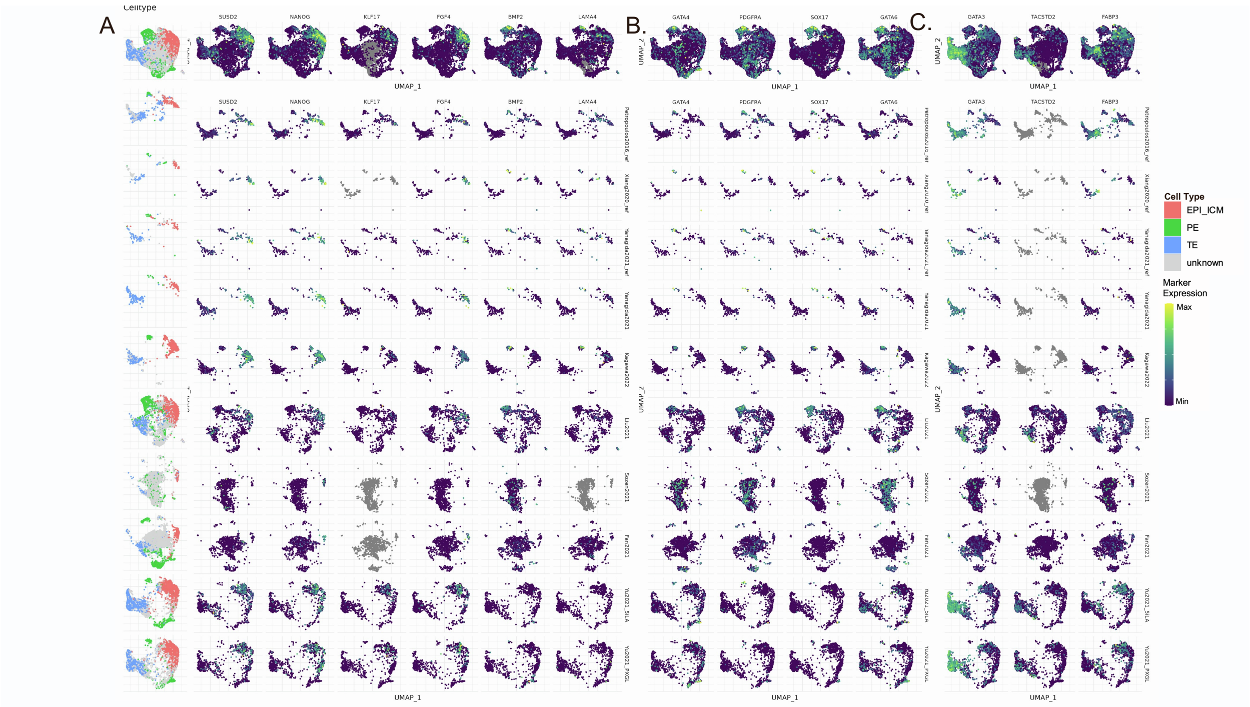

Figure S4. Preimplantation Marker Expression in Shared Gene Space, Related to Figure 2: Gene expression normalized by dataset is plotted in the shared gene space UMAP grouped according to the lineage, with each UMAP scaled independently for (A) EPI_ICM, (B) PE, and (C) TE.

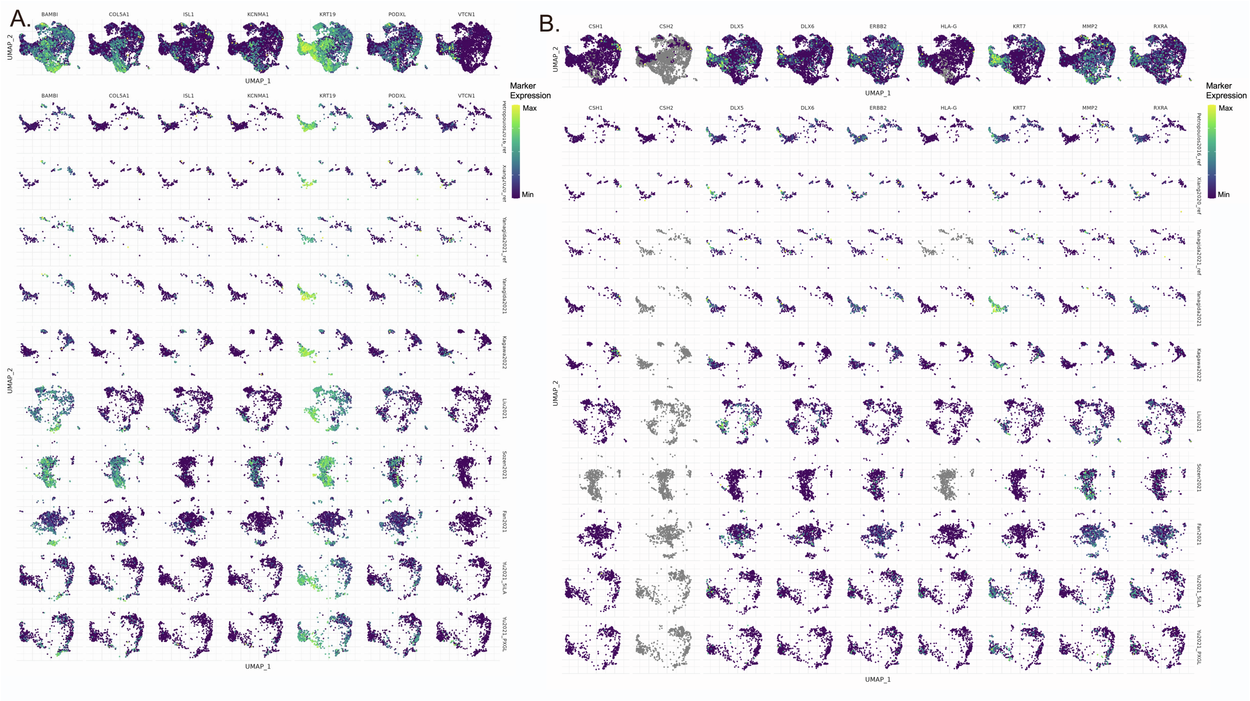

Figure S5. AMN and EVT Marker Expression in Shared Gene Space, Related to Figure 3: Gene expression normalized by dataset is plotted in the shared gene space. UMAP is grouped according to the lineage, with each UMAP scaled independently for the markers of (A) AMN and (B) EVT.

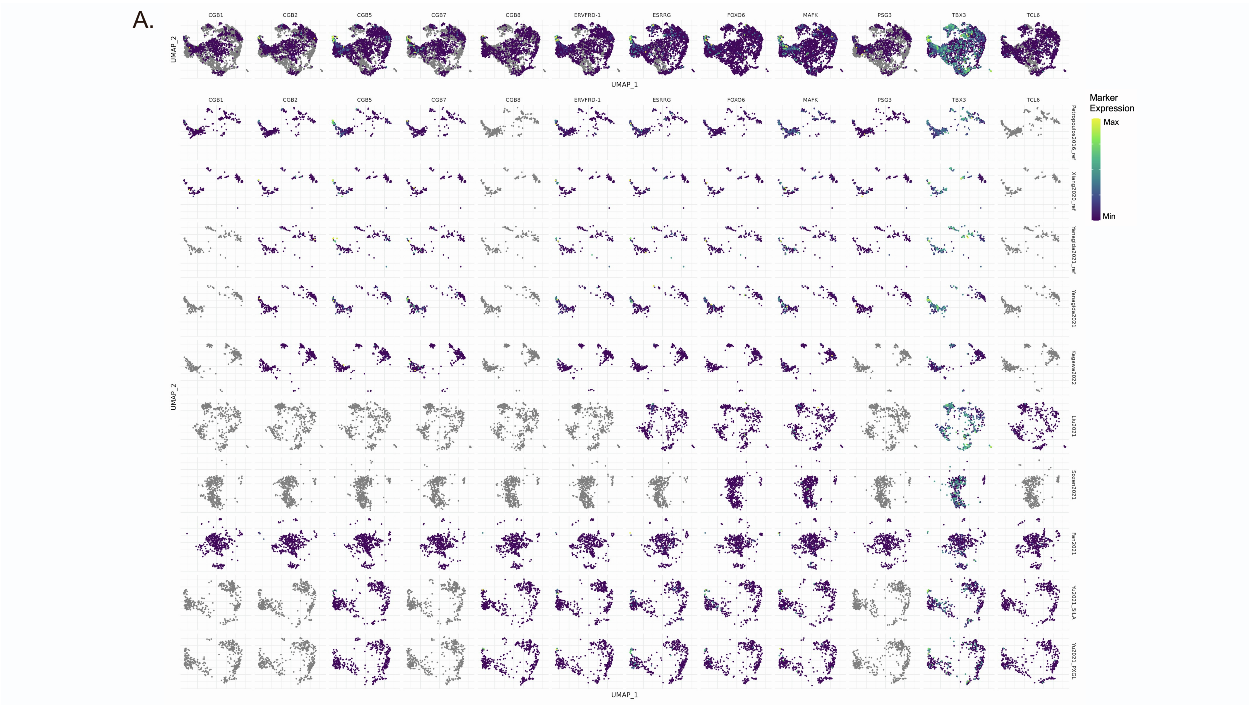

Figure S6. STB Marker Expression in Shared Gene Space, Related to Figure 3: Gene expression normalized by dataset is plotted in the shared gene space. The UMAP is scaled independently for each STB marker.

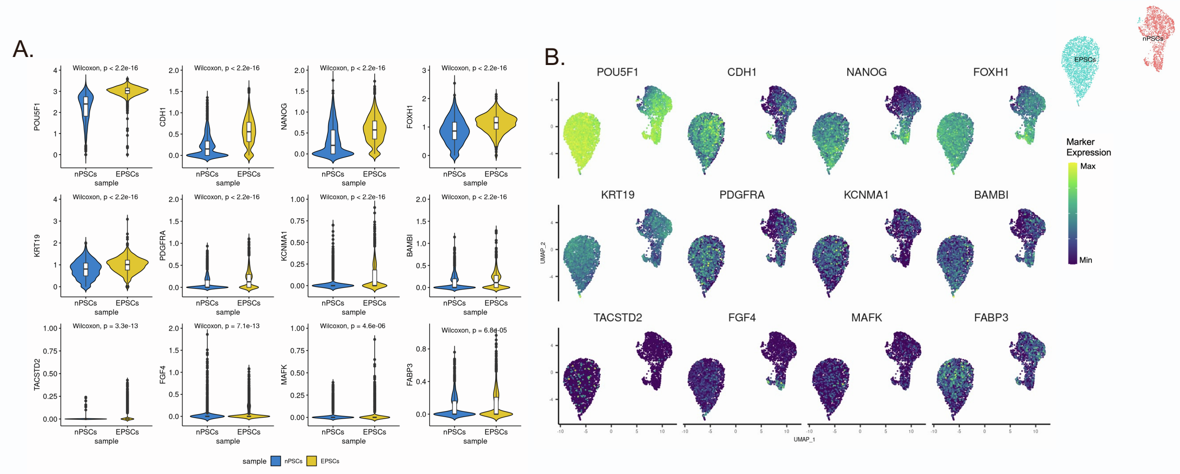

Figure S7. Marker Expression Between nPSCs and EPSCs, Related to Figure 5: A. The abundance of the lineage markers between the two founding cell lines along with wilcoxon rank-sum test p-value. B. UMAP plot of the lineage markers in the founding cell line with reference UMAP annotated by cell line.

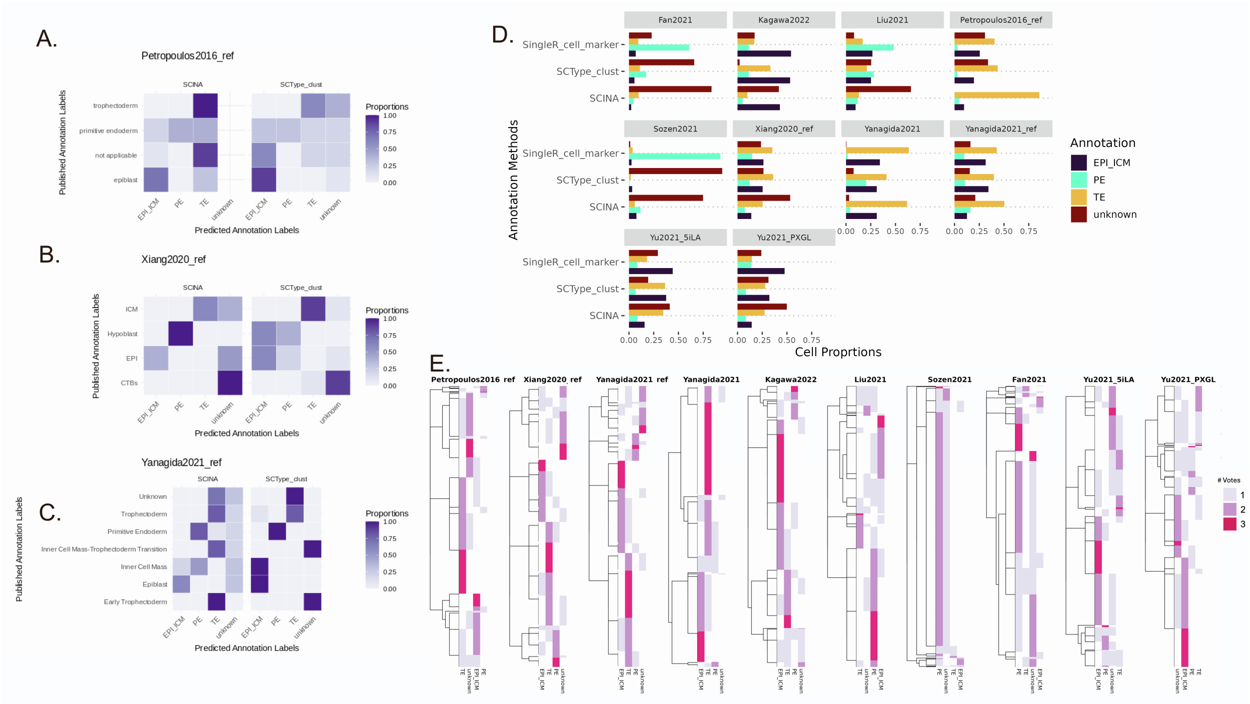

Figure S8. Annotation Validation and Quality Control, Related to STAR Methods: A. Contrast the assigned labels by marker-based annotation methods SCINA (left) and SCType (right) to the original publication labels for Petropoulos et al. 2016 blastocyst data[1]. B. Similar to A, but done for Xiang et al. 2020 ex vivo blastocyst data[2]. C. As in A and B, but for Yanagida et al., 2021 blastocyst data[3]. D. The frequency of the cell type labels generated by the different annotation methods across all datasets used in the analysis. E. The cell type number of votes heatmap for all cells in each dataset.

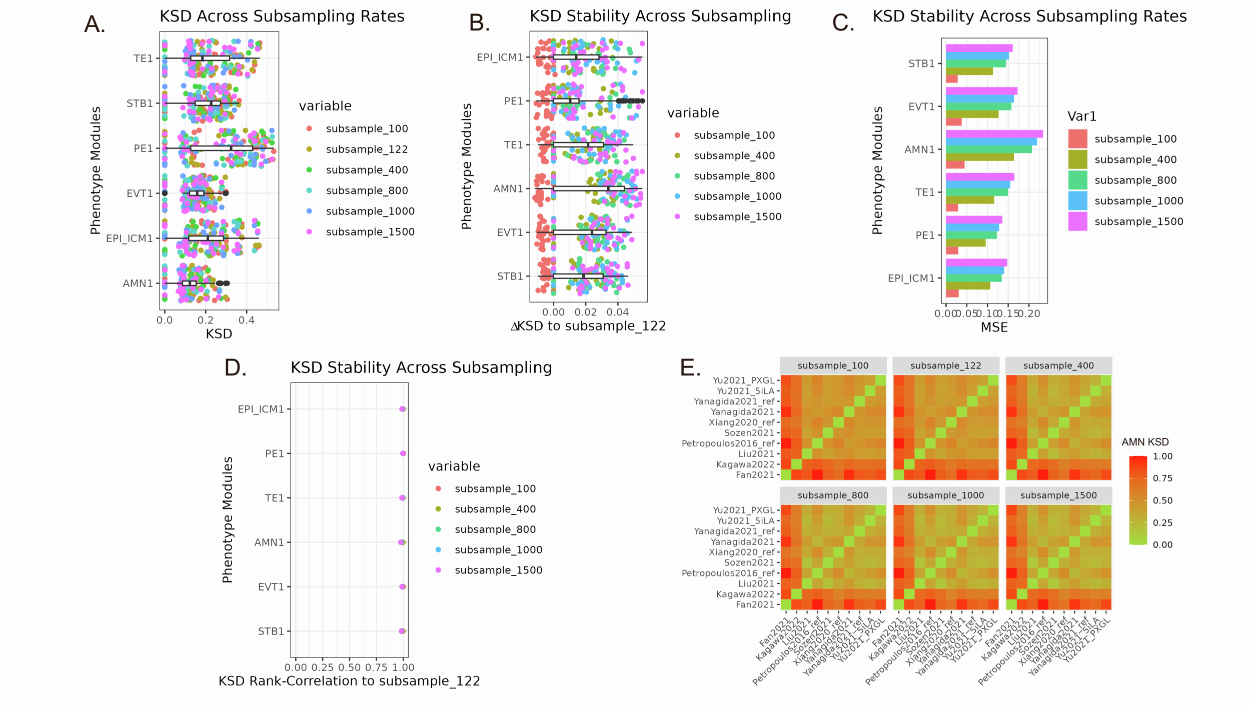

Figure S9. KS Distance Sensitivity Analysis, Related to STAR Methods: A. The lineage module score KD-distance for different sampling rates as indicated by the legend. B. The difference of each lineage module score KS distance between two dataset pairs with respect to 122 samping rate. C. Mean square error of the lineage module score KS distance matrices between each sampling rate and 122 samples (used in the study). D. Spearman rank-correlation between the different lineage module score KS distance matrices indicated for each sampling rate. E. Each heatmap shows the KS-distance (0 being identical to 1 being non-similar) between the AMN module scores across datasets (similar to the one shown in Figure 2D-E). Each panel shows the matrix computed using different subsampling sizes in the bootstrapping step.

References

1. Petropoulos, S., Edsgärd, D., Reinius, B., Deng, Q., Panula, S.P., Codeluppi, S., Plaza Reyes, A., Linnarsson, S., Sandberg, R., and Lanner, F. (2016). Single-Cell RNA-Seq Reveals Lineage and X Chromosome Dynamics in Human Preimplantation Embryos. Cell *165*, 1012–1026. https://doi.org/10.1016/J.CELL.2016.03.023.

2. Xiang, L., Yin, Y., Zheng, Y., Ma, Y., Li, Y., Zhao, Z., Guo, J., Ai, Z., Niu, Y., Duan, K., et al. (2020). A developmental landscape of 3D-cultured human pre-gastrulation embryos. Nature *577*. https://doi.org/10.1038/s41586-019-1875-y.

3. Yanagida, A., Spindlow, D., Nichols, J., Dattani, A., Smith, A., and Guo, G. (2021). Naive stem cell blastocyst model captures human embryo lineage segregation. Cell Stem Cell *28*, 1016-1022.e4. https://doi.org/10.1016/J.STEM.2021.04.031.
